# Toward the Optimization of the Optical Behavior of Transparent Wood: Current State of the Art and Perspectives

**DOI:** 10.3390/polym17243276

**Published:** 2025-12-10

**Authors:** Diego Pugliese, Giulio Malucelli

**Affiliations:** 1 Istituto Nazionale di Ricerca Metrologica (INRiM), Strada delle Cacce 91, 10135 Torino, Italy; d.pugliese@inrim.it; 2 Department of Applied Science and Technology, Politecnico di Torino, Viale Teresa Michel 5, 15121 Alessandria, Italy

**Keywords:** transparent wood, delignification, bleaching, resin infiltration, polymerization/curing, light scattering, refractive index, optical transmittance, haze

## Abstract

Transparent wood (TW) is a type of bio-based optical composite that combines wood’s hierarchical microstructure with polymers’ tailored optical properties to achieve high transmittance and controlled light scattering. TW is developed by removing lignin or modifying lignin chromophores and infiltrating a polymer whose refractive index closely matches that of the delignified wood framework. This review critically examines the parameters governing transparency in millimeter-thick TW, including the influence of wood species, delignification and bleaching strategies, and polymer selection for infiltration and polymerization/curing. The discussion emphasizes the interplay between microstructural anisotropy, refractive index matching, and processing-induced defects, which collectively determine light transmittance and haze. The review summarizes current progress toward achieving glass-like transparency in the millimeter range, highlighting the advances and remaining challenges in optimizing TW for scalable structural and functional applications.

## 1. Introduction

In materials science, transparency (also known as diaphaneity or pellucidity) represents the ability of a material to allow radiation of a certain wavelength (or in a range of wavelengths) to pass through it, limiting, as much as possible, its attenuation, scattering, reflection, or absorption [1,2]. Many materials can be considered transparent: very well-known examples comprise ceramics (i.e., glasses) and polymers. Their transparency is usually strictly related to the structure/morphology of these materials, where their constituents are not organized according to an ordered structure. In other words, transparent materials are usually amorphous. This condition allows the incident radiation to pass through them without undergoing deviation (i.e., avoiding scattering phenomena). Scattering is caused by the difference in density (and, therefore, in refractive index; *see* § 2.1) between the crystalline and amorphous regions in a semicrystalline material.

**Figure 1:**
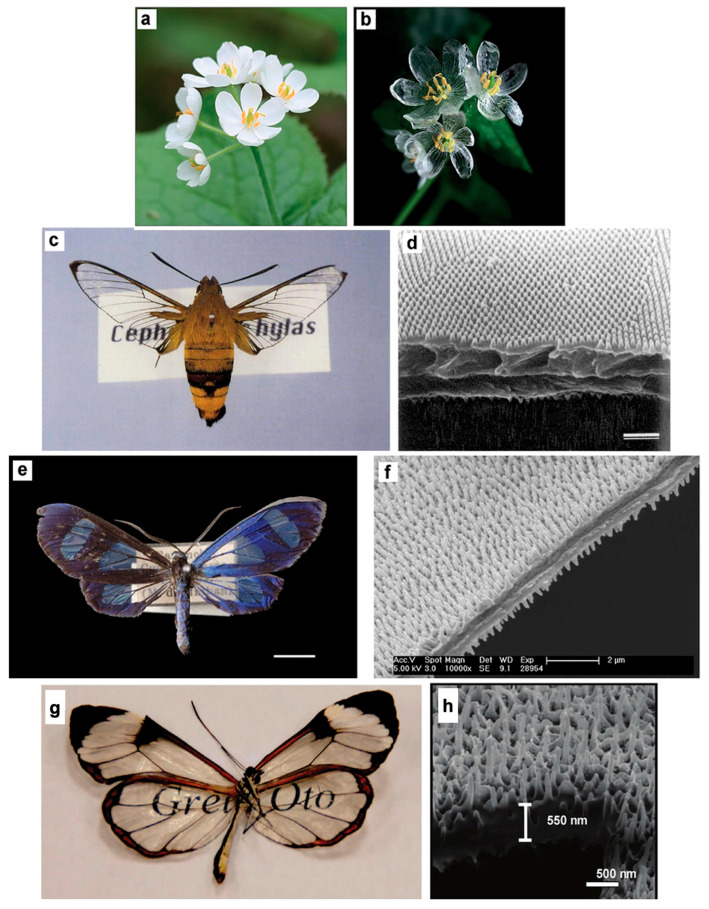
Examples of transparent materials in nature: photographs of the petals of *Diphylleia grayi* on a dry day (**a**) and on a rainy day (**b**); photograph of the hawkmoth *Cephonodes hylas* (**c**); scanning electron microscopy (SEM) image of the transparent part of the *Cephonodes* wing—the top side shows a regular-hexagonal packing of pillars (scale bar: 1 μm (**d**)); photograph of the moth *Cacostatia ossa* (scale bar: 0.5 cm (**e**)); SEM image of the wing of *Cacostatia ossa*: view of the wing surface at oblique incidence (**f**); photograph of a *Greta oto* butterfly (wingspan: about 47 mm (**g**)); cross-section of the wing membrane prepared by focused ion beam and imaged by SEM (**h**). Adapted from [3] under CC-BY-4.0 License.

Transparency is also common in nature (Figure 1), though naturally transparent materials are less often found than their opaque naturally occurring counterparts [3]. In particular, many minerals (such as quartz [4], diamonds [5], and several crystalline gemstones [6], among others) and animals (like jellyfish [7], glass frogs [8], glasswing butterflies [9], glass octopuses [10], and Antarctic ice-fish [11], among others) show certain transparency levels.

In addition, there exists a plethora of man-made materials that somehow mimic the transparency of naturally occurring counterparts [12]. As a few examples, unless transformed into glass-ceramics by nucleation and growth processes of ordered phases, glasses are undoubtedly amorphous and therefore transparent [13]. Conversely, some semi-crystalline polymeric materials (such as poly(4-methyl-1-pentene) [14] and poly(4,4′-aminocyclohexyl methylene dodecanedicarboxylamide) [15], among others), despite their partially ordered structure, are transparent because the densities of the amorphous and crystalline regions are practically the same and radiation therefore does not undergo scattering as it passes through them. In other words, the difference in refractive index between the amorphous and crystalline domains is negligible, and radiation scattering is completely avoided.

Wood is a unique, sustainable alternative to conventional, petroleum-based transparent polymers because it is renewable, has a hierarchical structure, and can fix carbon naturally. As a bio-based material, wood stores atmospheric carbon dioxide through photosynthesis, and can be exploited for different advanced applications [16,17,18,19,20]. This provides wood with a significantly lower environmental footprint than synthetic polymers, such as poly(methyl methacrylate) (PMMA), polycarbonate, and polyethylene terephthalate. Furthermore, wood’s hierarchically organized cellular microstructure naturally enables tunable optical and mechanical anisotropy when the lignin is removed or modified (bleached) and the so-obtained wood template is infiltrated with an index-matched monomer. Indeed, among the man-made transparent materials, transparent wood (TW) is gaining significant interest not only from academia but also from industry, thanks to its peculiar characteristics. Indeed, several comprehensive reviews have recently highlighted the potential of this new and intriguing material, addressing the research on this topic toward the development and implementation of more performing systems, cleaner technologies, and innovative potential structural and functional applications [21,22,23,24,25,26,27,28,29,30,31,32,33,34,35,36].

TW is able to combine high optical transmittance, mechanical robustness, and thermal insulation, making it attractive for sustainable optical and architectural applications [37]. In addition to its use in optical, photoelectric, and building materials, delignified or modified wood systems are being explored more and more for use in functional material systems, adsorbents, catalysts, fuels, and bio-based chemicals [21,23,26,27,28,30,31]. This extends their utilization value across multiple technological sectors and reinforces their relevance within the circular bioeconomy [22,38]. Recent studies have emphasized that TW’s combination of biogenic origin, optical tunability, and structural anisotropy makes it a pivotal platform that bridges the gap between optical functionality and environmental sustainability [31,34,39].

Very briefly, TW can be obtained either through lignin removal or lignin modification: the former strategy is based on the utilization of different chemicals (employed in alkaline or acidic conditions), which diffuse in the lignin-rich wood channels and extract the lignin contained therein. Conversely, considering that (i) lignin is the wood component mainly responsible for its mechanical strength, and (ii) the lignin removal may imply the use of quite toxic products and give rise to the formation of environmentally impactful by-products (e.g., sulfides and chlorinated compounds), lignin modification could be preferable. In this view, it was demonstrated that bleaching solutions comprising NaOH/Na_2_SO_3_/H_2_O_2_/diethylenetriaminepentaacetic acid can selectively remove the lignin chromophores, keeping more than 80% of the macromolecule within the wood channels, and, hence, providing transparency to the bleached material [40]. The successive step after removing lignin involves either compressing the material with an external pressure to densify the delignified cell walls or infiltrating the delignified wood with a suitable resin (this latter is the most preferred way; after infiltration, the resin undergoes polymerization/curing, depending on its thermoplastic/thermosetting characteristics). Though the final material’s flexibility is closely related to the variety of pristine wood and its initial thickness, compression generally allows for the fabrication of flexible TW thin films, which can be employed as they are or additionally vacuum-infiltrated by a suitable polymer resin. This way, also after a subsequent polymerization/curing step (in the presence of the infiltrated resin), it is possible to obtain transparent compressed wood materials [41,42].

However, regardless of the strategy adopted for the making of TW, the key point is to ensure a perfect match between the refractive index of the delignified/modified wood and the infiltrated resin system: this way, it is possible to remarkably limit the scattering phenomena of the incident radiation, providing the material with a high transparency.

**Figure 2:**
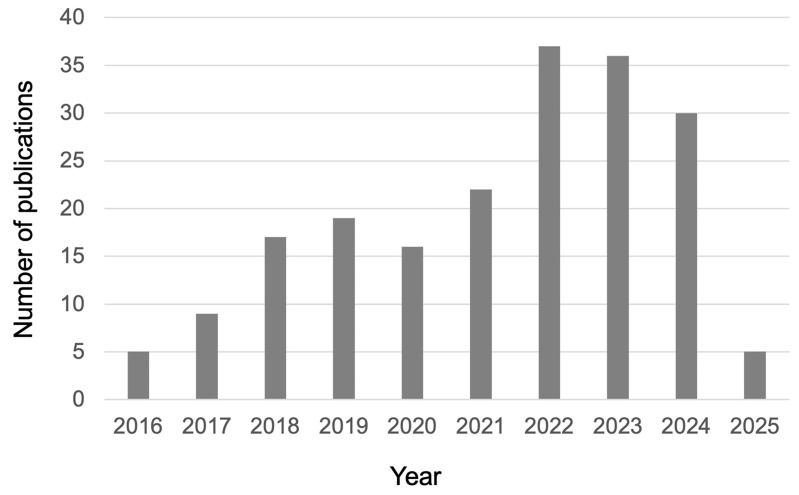
Number of publications (from 2016 to 2025) in peer-reviewed journals, dealing with “Optical properties AND transparent wood” (AND is the Boolean operator; data collected from the Web of Science™ database, accessed on 29 April 2025).

The research efforts that appeared in the scientific literature so far, specifically devoted to the investigation of the optical properties of TW (Figure 2), clearly indicate a wide interest in their development, implementation, and optimization.

Unlike previous reviews, which primarily focused on preparation techniques or the general performance of TW, this study provides a systematic analysis of the optical optimization of transparent wood within the millimeter thickness range. It emphasizes the quantitative relationships between composition, thickness, optical transmittance, and haze. Through a thorough examination of recent literature, this review clarifies how wood species, delignification and bleaching methods, and polymer infiltration strategies collectively impact light transmission and scattering. Particular attention is devoted to identifying the physical mechanisms that govern thickness-dependent transmittance and haze mitigation strategies, thereby outlining the key parameters that must be controlled to achieve glass-like clarity in thick TW structures.

Therefore, this work aims to review the most recent progress carried out so far in making wood transparent, discussing the effect of the various involved experimental parameters. Before addressing the discussion toward the optical outcomes in TW, a detailed overview about the assessment of transparency (or, more in general, about how to measure the optical properties of polymer systems) will be proposed, to provide the reader who is not familiar with the topic with the basic required concepts. Finally, the current limitations will be discussed and some research perspectives in the design and fabrication of TW with excellent optical features will be provided to the reader.

## 2. Evaluating the Optical Behavior of TW and Polymeric Systems

The main properties aimed at determining the optical behavior of TW, namely the refractive index (RI), the colorimetric response, the transmittance and the haze, are briefly defined in the following subsections. Their detailed description is reported in Section S1 of the Supplementary Materials.

### 2.1. Refractive Index

The RI is a fundamental optical property that quantifies how light propagates through a material. It is defined as the ratio of the speed of light in vacuum relative to the speed of light passing through that material. The RI of a material varies with the wavelength of light, a phenomenon known as dispersion, which is why precise values are generally specified for monochromatic light at a particular wavelength. Additionally, the RI is sensitive to temperature changes [43].

### 2.2. Colorimetric Response

The process of delignification and subsequent polymer infiltration in the fabrication of TW often leads to noticeable changes in its color characteristics. Colorimetry enables the quantitative assessment of these changes, particularly in terms of brightness, whiteness, and yellowing, which are critical for both aesthetic appeal and functional performance [44].

### 2.3. Optical Transmittance

Transmittance (*T*) refers to the overall quantity of light transmitted through a sample, encompassing both directly transmitted (ballistic) light and scattered light, and can be described by Equation (1) [45]:

(1) T=B+FS=I−A−R−BS,

where *B* is the number of ballistic photons passing through the sample, *FS* and *BS* are the amount of light forward and back scattered by the sample, respectively, *I* is the amount of the input beam, and *A* and *R* are the total amount of light absorbed and reflected by the TW sample, respectively.

### 2.4. Haze

Haze is a critical optical property of TW, often discussed alongside total transmittance, as it characterizes the degree of light diffusion and directly influences image clarity. Phenomenologically, haze represents the relative amount of forward-scattered or diffused light, excluding ballistic photons (*B*) that pass through the material without deviation, and leads to a decrease in image contrast when viewing objects through the sample [45]. It can be approximated as:

(2) $$Haze≅\frac{FS}{T},$$

where *FS* is the forward-scattered light and *T* is the total transmitted light. However, there is no universally accepted formal definition of haze, particularly regarding the divergence angle at which outgoing photons are still considered ballistic [24].

## 3. Transparency in Transparent Wood: Current Achievements

As some comprehensive reviews on TW are mainly focused on the preparation and overall behavior of the material, rather than specifically on its optical features [21,22,23,24,25,26,28,30,32,33,46,47,48,49,50,51,52,53,54,55,56,57,58], this section aims to summarize the key results obtained in the last four years, particularly evaluating how the type of native wood, the process of delignification or modification of the lignin, and the type and formulation of resins infiltrated into the wood may affect the overall optical features of the final material. For the reader’s simplicity, the discussion will be organized in the following Subsections by dividing it based on the type of native wood employed.

### 3.1. Balsa Transparent Wood

Balsa is one of the most commonly used native woods for producing TW [29].

Fan and co-workers [59] exploited a hydrogen peroxide solution in combination with UV radiation (employed as a catalyst) for oxidizing the chromophores of the lignin. Then, the modified balsa wood (2 mm thick) was infiltrated with a melamine formaldehyde resin containing a phosphorus-based flame retardant obtained through the reaction of a cyclic phosphate ester with low molecular weight (MW) poly(ethylene glycol). The optical properties of the final material were evaluated by measuring the optical transmittance, reflectance, absorption, and haze of the TW samples within the UV and Vis wavelength range. Despite lignin modification, the high difference in RI between air and the cell walls (1 vs. 1.53, respectively) accounted for a quite limited transmittance both in the longitudinal and transverse directions (i.e., respectively, parallel and perpendicular to the direction of wood growth), showing values below 60 and 50%, respectively. Conversely, optical transmittance values as high as 95 and 86% within 600 and 800 nm were obtained, respectively, in the longitudinal and transverse directions in the TW specimens. This finding was ascribed to the excellent matching between the RI of the infiltrated polymer and that of the wood cell walls after lignin modification. Furthermore, the TW exhibited a high haze level (approximately 96%), making it an ideal material for use in glazing applications, which provide uniform indoor lighting while ensuring privacy and visual comfort. Additionally, this high haze suggests that this TW is suitable for solar cells, which can boost the photoelectric conversion rate by enhancing the forward scattering of incident light.

**Figure 3:**
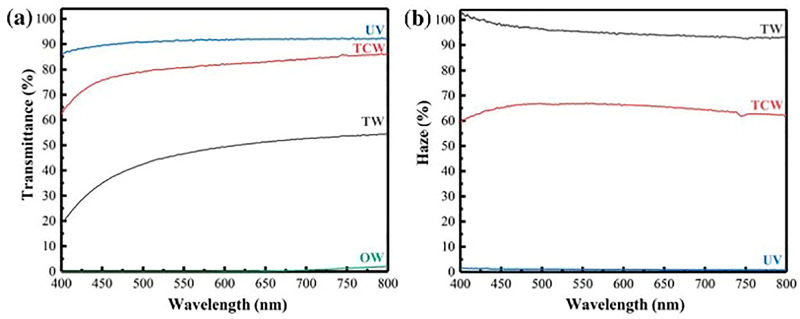
Characterization of the optical properties of balsa wood-derived samples: (**a**) Transmittance curves of native balsa (OW), UV-curable resin (UV), transparent wood (TW), and transparent compressed wood (TCW); (**b**) Haze curves of the same wood samples. Adapted with permission from [41]. Copyright Springer Nature, 2022.

Another interesting approach for enhancing the optical behavior of TW was proposed by Wang and co-workers [41], who first treated the native wood with NaClO_2_ in acidic conditions. Then, after densification of the cell walls by applying an external pressure, a delignified wood template was obtained. Finally, the template underwent a vacuum impregnation step with a UV-curable resin to obtain, after UV-curing, the final compressed TW (thickness of about 0.7 mm). The optical transmittance and haze were measured with a visible light photometer operating between 380 and 780 nm. The optical transmittance of the transparent compressed wood at 550 nm wavelength (Figure 3) was remarkably higher (about 81%) than that of the TW obtained using the same delignification, infiltration, and UV-curing procedures, but omitting the compression step (around 47%). This finding was attributed to the densification effect exhibited by the transparent compressed wood, in which the scattering phenomena were limited due to the reduced porosity of the wood template and, consequently, the entrapped air. This densification effect also decreased the haze, which changed from around 95 to 67% at 550 nm for TW and its compressed counterpart, respectively.

**Figure 4:**
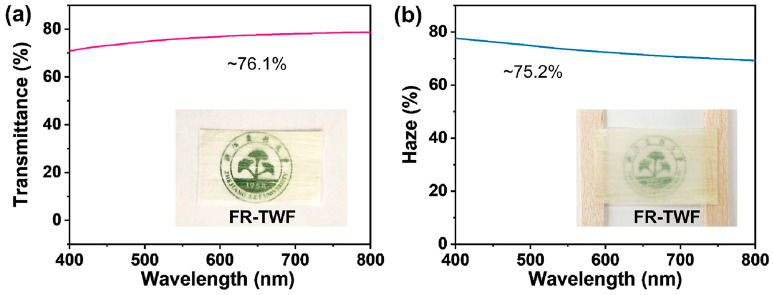
Optical properties of flame retardant transparent balsa wood samples (FR-TWF): (**a**) Transmittance and (**b**) haze curves. Adapted from [60] under CC-BY-4.0 License.

Zhou et al. [60] exploited a top-down chemical strategy for obtaining flame retarded TW. In particular, 2 mm thick balsa slices were first treated with sodium chlorite in acidic conditions and then went through a 2,2,6,6-tetramethylpiperidine-1-oxyl radical (TEMPO)-mediated oxidation treatment to complete the lignin removal and provide the wood channels with carboxyl groups, favoring the subsequent chelation with Al^3+^ cations. Finally, the resulting material underwent a self-densification/drying process, giving rise to a structure with fully collapsed cell walls and an elastic lumen. As shown in Figure 4, the optical transmittance and haze at 550 nm wavelength were approximately equal to 75%; this finding was ascribed to the collapsed cell walls that provided the TW with a uniform RI (approaching 1.53).

**Figure 5:**
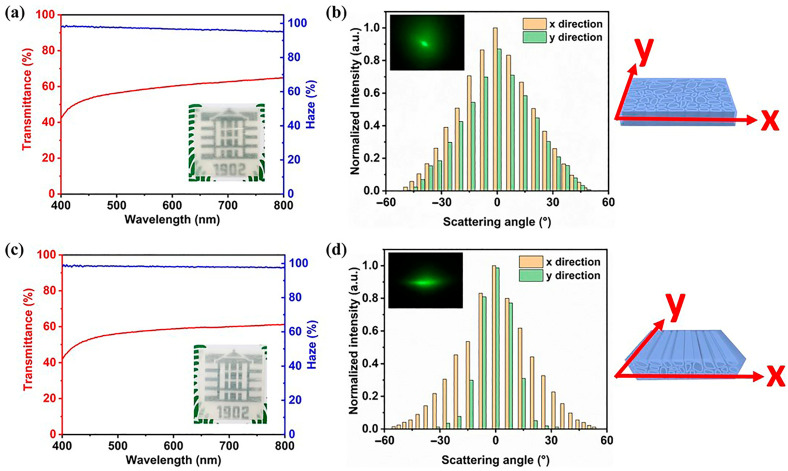
Optical properties of editable shape-memory TW: optical transmittance and haze of transverse (**a**), and longitudinal (**c**) TW; normalized intensity distribution of scattered light in the x- and y-directions for transverse (**b**), and longitudinal (**d**) TW, where the insets show photographs of their scattered light spots. Reprinted with permission from [61]. Copyright Springer Nature, 2022.

Wang and co-workers [61] succeeded in obtaining editable shape-memory TW materials exhibiting interesting optical features. To this aim, balsa wood slices (2 mm thick, taken from both longitudinal and transverse directions) were first treated in acidic NaClO_2_ solution, freeze-dried, and subsequently infiltrated with an epoxy vitrimer (i.e., bearing exchangeable covalent bonds), and cured. Optical transmittance and haze were evaluated using a light diffusion system equipped with an integrating sphere; in addition, the material’s light scattering was assessed with a green laser (wavelength: 532 nm) irradiating the samples at a 50 cm distance. The results are presented in Figure 5.

Both optical transmittance and haze were found independent from the cut direction of wood, and equal to 60 and 95%, respectively. Conversely, optical anisotropy was observed when the TWs were tested using the green laser: in fact, the transmitted light scattering revealed an anisotropic pattern for the longitudinal TW samples only, which showed larger angular distributions along the x-direction (that is perpendicular to the orientation of the wood channels) than those along the y-direction (that is parallel to the orientation of the wood channels). This finding was ascribed to the higher alignment of cellulose nanofibers along x-direction.

Wu et al. [62] delignified balsa wood (thickness: 1.2 mm) via acidified NaClO_2_ solution; meanwhile, they synthesized titania nanoparticles by in situ hydrolysis of tetrabutyl titanate and dispersed them in an epoxy resin, suitable for the infiltration process, at different loadings and in the presence of 3-glycidoxypropyltrimethoxysilane (used as a compatibilizer); finally, the infiltrated wood was cured. Haze and optical transmittance were measured using a scan UV–Vis–NIR spectrophotometer. Unlike the TW not embedding titania nanoparticles, which showed a limited transmittance (about 60%) in the visible wavelength range, the presence of increasing amounts of nanofiller accounted for a remarkable increase in the transmittance (up to 90%): this finding was attributed to the high RI of titania nanoparticles, which increased the low RI of the epoxy system, hence better matching with that of the delignified wood and increasing the optical transparency of the final material. Conversely, the incorporation of the nanofiller into the infiltrated epoxy did not affect the haze, which was around 90%.

**Figure 6:**
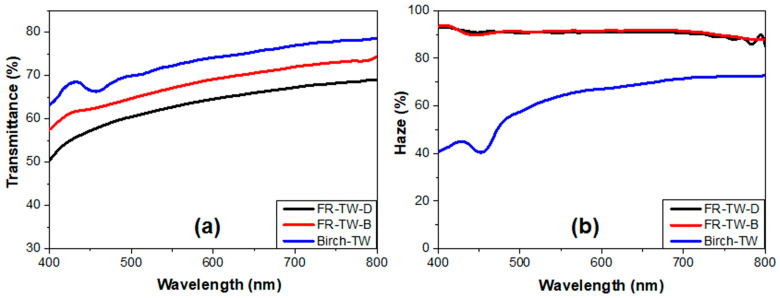
Optical transmittance (**a**) and haze (**b**) for balsa TWs. Legend: FR-TW-D = TW infiltrated with melamine-formaldehyde from delignified balsa; FR-TW-B = TW infiltrated with melamine-formaldehyde from bleached balsa; Birch-TW = birch TW infiltrated with PMMA. Reprinted from [63] under CC-BY 4.0 License.

Samanta et al. [63] applied bleaching or delignification to balsa wood veneer (typical size: 20 × 20 × 1 mm^3^). Bleaching was carried out using an aqueous solution including sodium silicate, sodium hydroxide, magnesium sulfate, hydrogen peroxide, and dimethylallyl triamine penta-acetic acid, operating at 70 °C until the treated wood achieved a white coloration. For the delignification of balsa wood veneer, an acetate buffer (pH around 4.6), including sodium chlorite, was used at 80 °C until the treated wood achieved a white coloration. Subsequently, the so-obtained materials were infiltrated with a flame-retardant, water-soluble melamine formaldehyde resin, cured at 150 °C for 15 min. Figure 6 shows the transmittance and haze of the obtained TWs, compared with those of a birch TW infiltrated with PMMA.

It is worth noticing that the TW obtained from bleached balsa wood, where lignin chromophores were removed, exhibited a higher optical transmittance than the counterpart from delignified balsa wood: this finding was ascribed to a reduction in such optical defects as voids and debond gaps, which promote light scattering. In addition, high haze values (around 90%) were measured within the investigated wavelength range for both types of balsa TWs. Finally, the scaling up of the production of larger transparent wood samples from bleached balsa (size of 200 × 100 × 1 mm^3^) did not affect the optical properties of the obtained materials: indeed, the overall optical performance of large-sized specimens was similar to that of small-sized counterparts.

**Figure 7:**
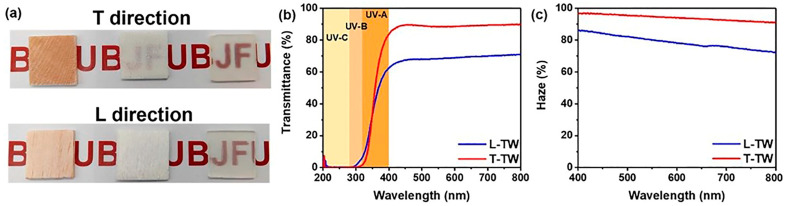
Optical properties of balsa TW infiltrated with a vitrimeric mixture: (**a**), from left to right, native wood pieces cut in the transverse (T) and longitudinal (L) directions, the related delignified materials, and the final TWs; (**b**) optical transmittance and (**c**) haze for the balsa TWs cut in the transverse (T-TW) and longitudinal (L-TW) directions. Adapted with permission from [64]. Copyright Elsevier, 2022.

Tan and co-workers [64] delignified 2 mm thick balsa wood (cut in both transversal and longitudinal direction with respect to the wood growth) by treating it at 80 °C in 1 wt.% NaClO_2_ aqueous solution for several hours; the pH of the solution was adjusted to 4.6 with glacial acetic acid. Then, the so-obtained wood was infiltrated with a vitrimeric mixture made of hexamethylene diisocyanate and tris(3-mercaptopropionate) (molar ratio between the two components: 3:2) and cured up to 60 °C. The resulting TW containing exchangeable thiocarbamate bonds showed multifunctional features (i.e., outstanding mechanical behavior in the longitudinal direction, with flexural and tensile strengths of about 49 and 37 MPa, respectively, thermal conductivity as low as 0.30 W⋅(mK), and shape reprocessability and restorability). In addition, as far as the optical properties of the final material are considered (Figure 7), high transmittance and haze over the visible wavelength range were observed (both achieving about 90% at 800 nm) for the TW cut in the transverse direction. At the same time, lower values of the two parameters were measured in the longitudinally cut counterparts (around 71 and 72% at 800 nm, respectively). This finding was attributed to a discrete RI variation along the longitudinal direction, which was promoted by the highly aligned cellulose nanofibers in the perpendicular direction (z-axis), hence resulting in an anisotropic scattering of transmitted light in the longitudinally cut samples.

To obtain a transparent and fireproof wood, Chu et al. [65] infiltrated delignified balsa wood with phosphate ester-polyethylene glycol. Delignification was carried out by soaking the native wood in a 2.5 M H_2_O_2_ solution for 48 h. The infiltrated monomer was then cured, and the performance of the final TW was compared with the same material infiltrated with an epoxy monomer (i.e., diglycidyl ether of bisphenol-A). Apart from excellent flame retardant features (i.e., self-extinction in horizontal flame spread tests and limiting oxygen index values as high as 37%), the transmittance of the TW infiltrated with phosphate ester-polyethylene glycol was around 93% within the visible wavelength range, i.e., higher than the corresponding counterpart infiltrated with the epoxy monomer (about 82%). Furthermore, the high haze of the TW infiltrated with phosphate ester-polyethylene glycol (around 98% in the visible wavelength range) suggested its potential suitability for civil engineering applications, e.g., for glazing and roofing components.

**Figure 8:**
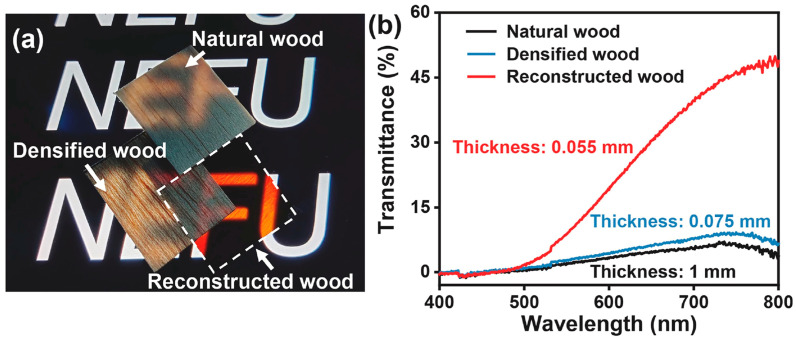
(**a**) Optical images of natural wood, densified wood, and reconstructed wood are shown to demonstrate the better visible transparency of reconstructed wood compared to the other two. (**b**) Visible transmittance spectra of the three materials. Adapted with permission from [66]. Copyright Elsevier, 2022.

Interestingly, Sun and co-workers [66] carried out a 24 h treatment of 1 mm thick balsa wood slices in 1 wt.% NaClO_2_ solution buffered at pH = 4.6 with acetic acid, at 80 °C. Then, the delignified wood underwent TEMPO-mediated oxidation and was subsequently infiltrated with commercially available alkali lignin, resulting in the formation of “reconstructed” TW. Compared with densified natural wood (obtained through hot-pressing native wood previously soaked in acetone), the reconstructed wood showed a much higher optical transmittance (with a maximum of about 48% at 800 nm; see Figure 8): this finding was attributed to its more densely packed structure, due to the alkali lignin infiltration.

**Figure 9:**
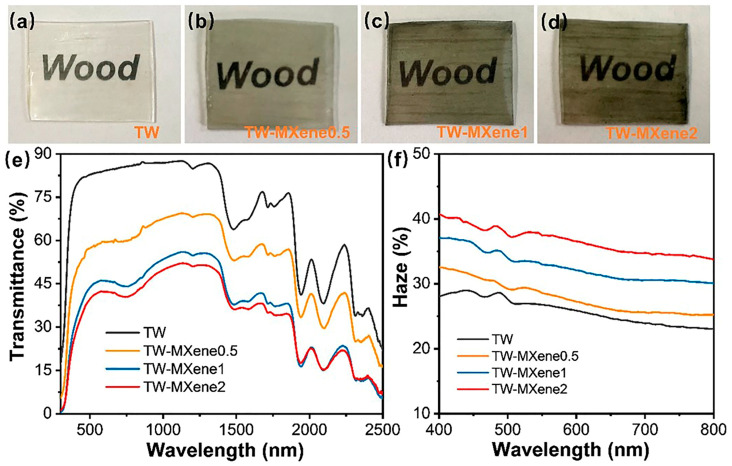
Typical pictures of transparent wood infiltrated with poly(vinyl alcohol) (TW) and with poly(vinyl alcohol) embedding different amounts of MXene nanosheets (**a**–**d**). Optical transmittance (**e**) and haze (**f**) of the investigated materials. Legend: TW-MXene0.5 = TW infiltrated with poly(vinyl alcohol) embedding 1 wt.% of MXene nanosheets; TW-MXene1 = TW infiltrated with poly(vinyl alcohol) embedding 2 wt.% of MXene nanosheets; TW-MXene2 = TW infiltrated with poly(vinyl alcohol) embedding 3 wt.% of MXene nanosheets. Reprinted with permission from [67]. Copyright Springer Nature, 2022.

Zhang and co-workers [67] delignified balsa wood slices (thickness: 1 mm) by using a 2 wt.% solution of NaClO_2_, buffered with glacial acetic acid at pH = 4.6, at 80 °C for 48 h. Then, poly(vinyl alcohol) incorporating 1, 2, or 3 wt.% of delaminated Ti_3_C_2_T_x_ (MXene) nanosheets was infiltrated into the delignified wood after it underwent freeze-drying for 48 h. As shown in Figure 9, increasing amounts of MXene nanosheets accounted for color darkening, as well as the decrease in the optical transmittance and increase in the haze of the resulting TWs. This finding was ascribed to the increasing absorption and scattering of the incident radiation because of the increase in the RI gap between poly(vinyl alcohol) and cellulose (due to the incorporated nanofiller), which promoted the occurrence of broader scattering phenomena at the interface between the polymer and the wood channels.

Ding et al. [68] delignified balsa wood (2.5 mm thick) with 2.5 wt.% NaClO_2_ solution buffered at pH = 3.5 with acetic acid. The process was carried out at 70 °C for 24 h. Then, the delignified wood (about 2.7% lignin content) was infiltrated with a bisphenol A diglycidyl ether-based epoxy system, cured, and, finally, coated with a perfluorodecyltriethoxysilane coating. The latter provided the TW with high hydrophobicity, achieving water contact angles of about 113°. Additionally, the resulting TW exhibited acceptable optical transmittance and haze of around 67 and 73%, respectively, at 750 nm.

An interesting approach for obtaining a multifunctional TW was recently proposed by Wang and co-workers [69], who designed and developed a shape-reconfigurable, thermally insulating TW through the infiltration of polythiourethane vitrimers into delignified wood (thickness: 1 or 2 mm). The latter was obtained through a treatment with 1 wt.% NaClO_2_ aqueous solution (keeping the pH at 4.6 with acetic acid, working at 80 °C, and replacing the solution every 6 h). The optical transmittance of the obtained TW was about 87 and 82%, respectively, for specimens 1 and 2 mm thick. Further, the haze referred to the same thicknesses was quite high, i.e., around 73 and 89%, respectively. These results were ascribed to the microstructural roughness of the final material.

Shi et al. [70] were among the first to provide further sustainability to TW. To this aim, balsa wood (0.8 mm thick) was first treated with a 5 wt.% NaClO_2_ solution in acidic conditions (keeping the pH at 4.6 through the addition of acetic acid), at 90 °C. Then, the obtained delignified wood was further treated with 30 wt.% hydrogen peroxide solution at 90 °C for 1 h. Meanwhile, phenol was reacted with glucose, both being prepared through the catalytic degradation of the lignin and cellulose, respectively. Then, the obtained polymer was infiltrated into the delignified and bleached balsa wood operating under vacuum at room temperature and finally cured. The so-obtained TW showed a limited optical transmittance, achieving around 46% at 800 nm. This finding was attributed to the porosity resulting from the delignification process, which was not thoroughly saturated with the infiltrated polymer, probably due to interface incompatibility issues between the polymer and the delignified wood channels.

**Figure 10:**
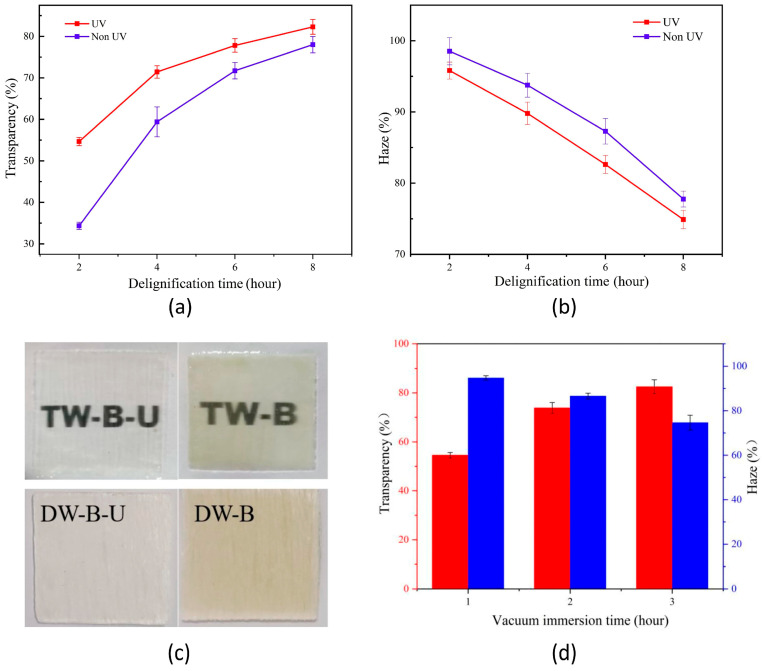
Optical transmittance (**a**) and haze (**b**) of TW samples obtained by dipping the native wood in a lignin-modifying solution under UV-assisted (UV) or dark conditions (Non UV) for 2, 4, 6, and 8 h, followed by a 3 h infiltration treatment with an epoxy system. (**c**) Photographs of the obtained TWs under daylight source. (**d**) Optical transmittance (red bars) and haze (blue bars) of TWs as a function of the infiltration time under vacuum. Legend: DW-B: delignified wood under dark conditions; DW-B-U: delignified wood under UV-assisted conditions (4 h exposure); TW-B: TW obtained from wood delignified under dark conditions; TW-B-U: TW obtained from wood delignified under UV-assisted conditions (4 h exposure). Reprinted from [71] under CC-BY-4.0 License.

Chen et al. [71] demonstrated that UV radiation can be successfully exploited for speeding up the lignin removal from balsa wood. For this purpose, 1 mm thick native wood samples were immersed into a solution consisting of H_2_O_2_, ammonia, and deionized water (keeping a ratio of 5:1:5 among the three components) for different times (from 2 up to 8 h), under continuous UV irradiation provided by a UV lamp (30 W) emitting between 385 and 395 nm. As reference materials, another set of samples underwent the same treatment in the dark (i.e., without having been UV-irradiated). The delignified materials were, then, infiltrated with an epoxy system under vacuum for 3 h and finally cured at room temperature for 1 day. As expected, the optical transmittance was remarkably affected by the duration of the delignification process. In this context, UV exposure accounted for accelerating the removal of the chromophore groups of lignin, hence leading to an overall improvement of the optical transmittance of the obtained TW (Figure 10). Interestingly, the optical transmittance increased with the infiltration time, while the haze gradually decreased, resulting in better optical properties. These findings were attributed to a decrease in the porosity in the wood channels infiltrated by the epoxy system.

**Figure 11:**
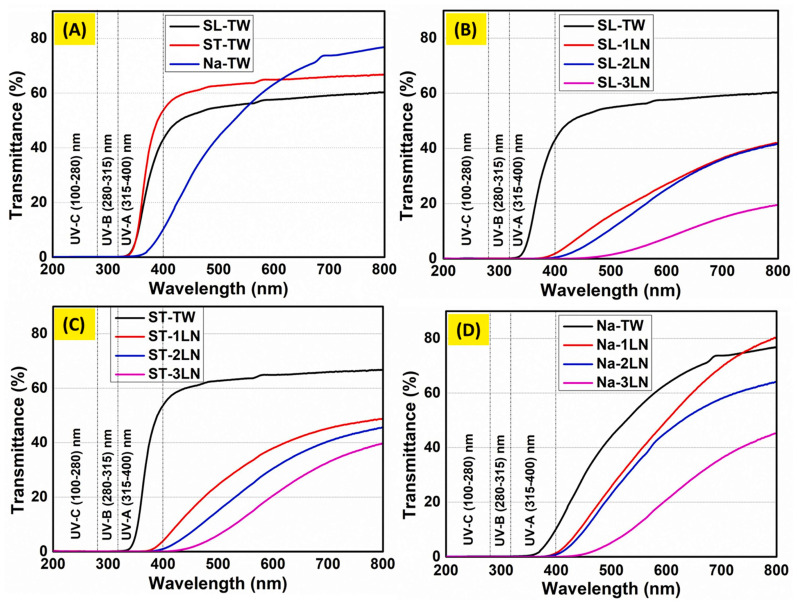
Optical transmittance of TWs (**A**) and their composites containing 1–3 wt.% of lignin nanoparticles (**B**–**D**). Legend: SL-TW: TW derived from solar-assisted bleaching; ST-TW: TW derived from steam bleaching; Na-TW: TW derived from NaOH delignification; SL-xLN: TW derived from solar-assisted bleaching and containing x wt.% of lignin nanoparticles; ST-xLN: TW derived from steam bleaching and containing x wt.% of lignin nanoparticles; Na-xLN: TW derived from NaOH delignification and containing x wt.% of lignin nanoparticles. Reprinted with permission from [72]. Copyright Elsevier, 2023.

In a further research effort toward sustainability, Hai et al. [72] proposed three different methods for the delignification of balsa wood (1 mm thick), namely: solar-assisted bleaching (employing H_2_O_2_/NaOH as bleaching agents and exposing the treated wood to direct sunlight), steam bleaching (treating the native balsa wood at 90 °C for 3.5 h with a H_2_O_2_/NaOH solution, also deposited onto the wood surface), and NaOH delignification (carried out at 90 °C for 6 h). Meanwhile, kraft lignin was converted into lignin nanoparticles, which, in turn, were incorporated into a poly(vinyl alcohol)/propylene glycol mixture at different loadings (i.e., 1, 2, and 3 wt.%), vacuum-infiltrated in the delignified wood and finally dried. As shown in Figure 11, the method employed for the delignification step remarkably affected the optical transmittance of the resulting TWs. In particular, within 600 and 800 nm wavelength, the material derived from NaOH delignified balsa wood exhibited an optical transmittance as high as about 80%, unlike the counterparts obtained through solar-assisted or steam bleaching, for which the optical transmittance was between about 60 and 65%. Further, the incorporation of increasing amounts of lignin nanoparticles accounted for a progressive decrease in the optical transmittance: this finding was attributed to the high RI of the nanoparticles (around 1.61), which promoted light absorption/scattering phenomena.

Chen et al. [73] delignified wood slices (1 mm thick) by treating them in an acidic solution (pH = 4.6) of sodium chlorite, working at the boiling point of the solution for 2 h. Then, the resulting delignified wood underwent an oxidation process in a 1 wt.% NaIO_4_ solution at 50 °C for 4 h. Subsequently, the dialdehyde-oxidized wood experienced a densification process at 60 °C for 48 h, after which it was laminated to obtain a three-ply transparent material. This material, which was about 3 mm thick, exhibited very low haze (around 20%) and high transmittance (approximately 85%) within the visible wavelength range. This is thanks to the material’s limited porosity (around 8%), which was achieved through the oxidation process of the wood cellulose. This process decreased the scattering centers (i.e., air voids) present in the laminate.

Yin et al. [74] succeeded in manufacturing a white luminescent TW, exploiting the incorporation of graphite carbon nitride (synthesized on purpose) into an epoxy system that was infiltrated in the delignified wood. The latter was obtained by treating the wood slices (2 mm thick) in a 5 wt.% NaOH solution at 90 °C for 2 h. The final curing of the infiltrated epoxy system was carried out at room temperature for 72 h. The optical transmittance of the final material exceeded 80% within the wavelength range of 500 to 800 nm. Additionally, the very high haze (approximately 82% within the same wavelength range), combined with the luminescent features, suggested the potential suitability of the material for designing photoelectric devices.

The optical features of TW were recently exploited for the design of thermochromic materials for smart windows [75]. In particular, balsa wood slices (1.5 mm thick) were rotary cut, subsequently brushed with hydrogen peroxide and sodium hydroxide, and finally exposed to UV radiation for 20 min. This process was repeated about 10 times, which was the amount of time needed to deactivate the lignin chromophores sufficiently. Then, the so-obtained material was vacuum-infiltrated with pre-polymerized methyl methacrylate for 24 h; the final polymerization took place in an oven at 70 °C for 4 h. In addition, to provide thermochromic features to the TW, its surface was spin-coated with a layer of MA_4_PbI_5_Br_1_·2H_2_O halide hybrid perovskite precursor (middle layer) and a further layer of PMMA (top layer). Within the visible wavelength range, in the cold state, the optical transmittance was around 78%; it dropped down to about 25% in the hot state, hence demonstrating the suitability of the materials for the design of thermochromic devices.

**Figure 12:**
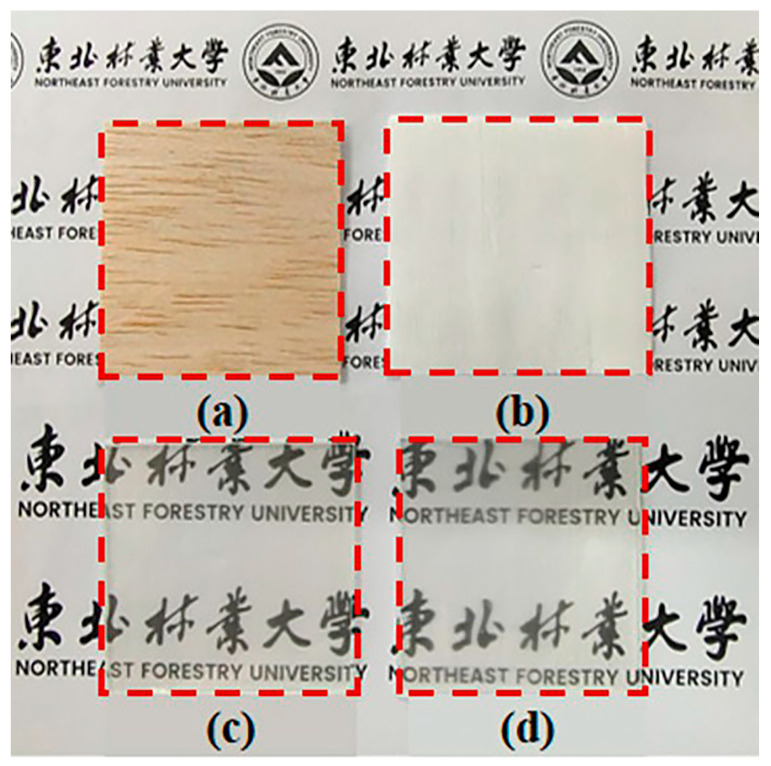
Typical photographs of pristine balsa wood (**a**), wood after the removal of lignin chromophores (**b**), TW (**c**), and hydrophobic TW (**d**). Reprinted from [76] under CC-BY-4.0 License.

A similar approach was proposed by Wang and co-workers [76], who exploited an effective and sustainable approach for obtaining a hydrophobic TW. For this purpose, they employed a UV-assisted method for the in situ removal of the lignin chromophores: native balsa wood (1.5 mm thick) first was treated with a mixture consisting of hydrogen peroxide, ammonia, and deionized water (7:1:3 volume ratio) under exposure to UV radiation for 4–6 h. Subsequently, after a drying step, the treated wood was infiltrated under vacuum with an epoxy system and cured. The obtained TW was, then, dipped in a polydimethylsiloxane-hexane solution for 10 min and cured in a vacuum drying oven at 80 °C for up to 4 h. Apart from the high hydrophobicity achieved after the final treatment with polydimethylsiloxane (as witnessed by the very high static contact angle values with water, around 130°), as shown in Figure 12, even the non-hydrophobic TW exhibited a high transparency. Moreover, the average optical transmittance and haze in the wavelength range of 400–700 nm were about 90 and 80%, respectively, hence indicating the potential suitability of the material for solar cells, smart windows, and energy-efficient building applications.

Wu et al. [77] produced a three-layer TW exhibiting thermal energy storage function. To this aim, a core made of delignified balsa wood vacuum-infiltrated with polyethylene glycol (average molecular mass: 1000 g/mol)-silica for 2 h and subsequently freeze-dried for 48 h was stacked within two delignified balsa wood layers infiltrated with methyl methacrylate (successively thermally polymerized). For the delignification step, the wood slices were treated with a 2 wt.% sodium chlorite solution, maintaining the pH at 4.6 with glacial acetic acid and operating at 85 °C, until the wood turned white. The multilayered final material (about 2.2 mm thick) exhibited 45 and 76% of optical transmittance and haze, respectively, at 800 nm wavelength, as well as a very homogeneous distribution of the scattered light, notwithstanding important heat storage capacity (with a latent heat as high as 15.0 J/g) and thermal stability.

The possibility of combining good optical transmittance with luminescent features in TW was assessed by Zhou and co-workers [78]. They first synthesized carbon quantum dots exhibiting yellow/red fluorescence from chitosan and o-phenylenediamine. The obtained material was then dispersed into a diglycidyl ether of bisphenol A-based epoxy system at a concentration of 10 g/L, vacuum-infiltrated into delignified wood for 30 min, and finally cured. For the delignification step, 1 mm thick balsa wood slices were treated with a 2 wt.% solution of sodium chlorite, keeping the pH constant at 4.6 by means of glacial acetic acid, and working at 80 °C for around 6 h. The red fluorescent TW exhibited good optical transmittance (about 70%) in the visible wavelength range, as well as effective UV blocking features. Specifically, it blocked approximately 79% of UV-B radiation and 78% of UV-A radiation.

Chen et al. [79] succeeded in obtaining TW films by exploiting a multistep process comprising: (i) the delignification of native wood slices (starting thickness: 1 mm) treated at 80 °C for 6 h with a mixture (equal volume) of glacial acetic acid and H_2_O_2_; (ii) the subsequent infiltration of the delignified wood in an aqueous solution of an ionic liquid (namely, 1-ethyl-3-methylimidazolium acetate, employed at 80 wt.% concentration) under vacuum at room temperature; (iii) the removal of the ionic liquid and the drying under vacuum in an oven at 60 °C for 4 h to attain capillary force driven self-densified TW films. The latter (around 150 μm thick) exhibited an optical transmittance and haze of about 70 and 95%, respectively.

In a further research effort toward sustainability, Zhou and co-workers [80] first delignified native wood slices (starting thickness: 1 mm) by treating them in a 2 wt.% sodium chlorite solution buffered at pH 4.6, for 2 h at the boiling point of the solution. Then, hemicellulose was largely removed by dipping the delignified wood in 15 wt.% sodium hydroxide solution for 2 h, at 40 °C. The next step was to oxidize the obtained material using a 1 wt.% NaIO_4_ solution at 50 °C for 4 h. Then, the modified wood was densified, grafted with a 0.1 wt.% gelatin solution at 60 °C for 4 h, and physically crosslinked with tannic acid at room temperature for 2 days. The densified TW (approximately 0.1 mm thick and fully biodegradable) exhibited high optical transmittance (around 86% at 600 nm), low haze (approximately 17% across the entire visible spectrum), and effective 100% UV-B blocking.

One example of thick TW showing interesting optical behavior was proposed by Liu and co-workers [81]. To this aim, a 1 wt.% NaClO_2_ aqueous solution was prepared and subsequently adjusted to a pH of 8.5 using glacial acetic acid. The wood slices (2 mm thick) were immersed in the delignification solution at a temperature of 80 °C for a period of 18 h. The delignification solution was replenished every 6 h, and the slices were left to soak until they had thoroughly changed color from their original shade to white. Then, a mixture of epoxy monomer (unspecified) and trimethylolpropane tri (3-mercaptopropionic acid) ester was employed for the infiltration process, working under vacuum, at room temperature, for 20 min, and finally cured at 120 °C for 2 h. The obtained TW exhibited soft-hard switchable behavior due to its low glass transition temperature (around 0 °C), as well as shape recovery features. Intriguingly, despite its considerable thickness, its optical transmittance averaged 70% in the visible wavelength range.

In a very recent paper, Wu and co-workers [82] demonstrated the feasibility of preparing multifunctional (i.e., UV-shielding and thermally insulated) TW samples with high optical transmittance (around 90%) and low haze (about 55%) in the visible wavelength range. To this aim, wood slices (5 × 5 cm^2^; the thickness ranged from 0.5 to 2 mm) were first immersed in an aqueous peroxyacetic acid solution with a pH of 4.8, adjusted using sodium hydroxide, at a solid-to-liquid ratio of 1:10 and at 80 °C. After about 2–4 h, the wood chips turned white, indicating the completion of the delignification process. Then, the delignified wood was immersed in a mixture of pre-polymerized methyl methacrylate and 4-vinylphenyl boronic acid (mass ratio up to 8:100) under vacuum for 30 min. Finally, it was cured under UV radiation for 1 h.

Table 1 summarizes the main optical outcomes related to the different TW systems discussed in the section.

### 3.2. Pine Transparent Wood

**Figure 13:**
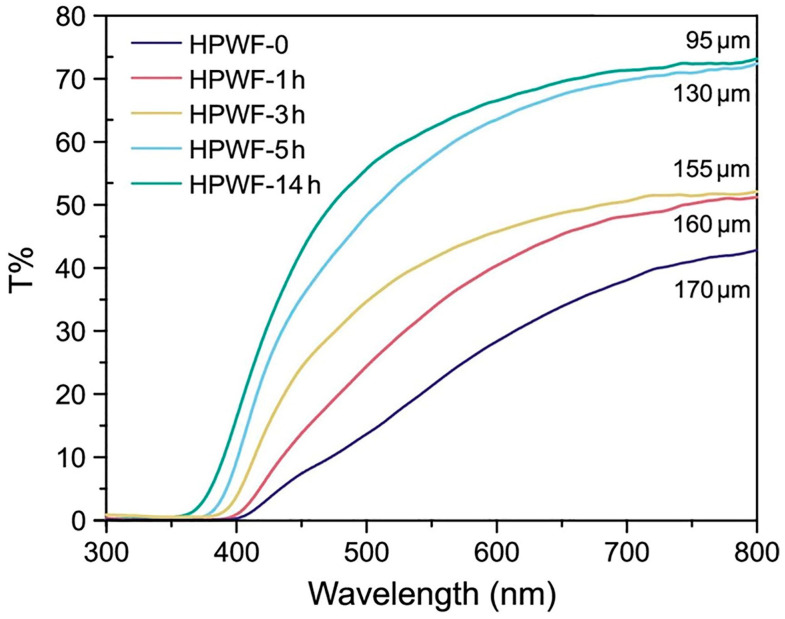
Optical transmittance of sulfonated, densified TW slices. Legend: HPWF-0 = native wood subjected to densification only; HPWF-Xh = sulfonated and densified pine wood, where X stands for the duration of the sulfonation process. Adapted from [83] under CC-BY-4.0 License.

A simple and interesting method involving in situ sulfonation, followed by hot pressing, for pine veneers, was developed by Mastantuoni et al. [83]. The goal was to prepare high-density, transparent, thin films preserving both wood components and pristine fiber alignment. To achieve this, Scots pine veneers (750 µm thick) were first immersed in a 0.7 M Na_2_SO_3_ solution; they underwent an initial impregnation step at 80 °C, followed by treatment at 165 °C for different durations (i.e., 1, 3, 5, and 14 h). The pH was kept at 7 using sulfuric acid. Then, the sulfonated veneer slices were pressed at room temperature under 250 kPa for 30 min, further pressed at 500 MPa at room temperature for 10 min, and finally treated at 150 °C for 30 min. As shown in Figure 13, the sulfonated, densified TW exhibited a high UV-blocking effect and acceptable optical transmittance. The transmittance decreased as the thickness increased.

Wang and co-workers [84] exploited a lamination process for obtaining multilayered TW. To this aim, scotch pine slices (0.2 mm thick) first underwent delignification by treating them in a 3.5 wt.% NaClO_2_ solution, keeping the pH at 4.6 with acetic acid and working at 85 °C for up to 3 h. Then, the delignified wood slices were infiltrated with a low MW bisphenol A epoxy resin mixed with a hardener (i.e., a polyetheramine) under vacuum and at room temperature. Finally, the infiltrated slices (three or five) were laminated under pressure at room temperature until curing was complete. The optical transmittance of the obtained laminates was strictly related to the number of TW layers present in the structure: in particular, the transmittance of 3-layer assemblies was in between 45 and 65% in the visible wavelength range, while it dropped to 35–55% in 5-layer assemblies.

Wang et al. [85] demonstrated the effectiveness of chemical crosslinking pretreatment in maintaining the integrity of naturally aligned pine wood fibers after delignification. First, the wood slices were immersed in a solution of 1,4-butanediol diglycidyl ether and water, while degassing under vacuum for 20 min at room temperature. Then, the crosslinking reaction was performed at 80 °C for 2 h with mild stirring. Next, the resulting material was treated with a 4 wt.% peracetic acid solution at a pH of 4.8 to delignify it. This process was carried out at 85 °C for 45 min. Finally, the wet crosslinked delignified wood veneer was pressed under 0.1 MPa at room temperature for 10 min to remove excess water. Then, the temperature gradually increased from room temperature to 105 °C. The sample was hot-pressed at 105 °C under 15 MPa for 30 min, producing transparent, naturally aligned holo-fiber films that were 150 µm thick. These films exhibited high optical transmittance and haze (approximately 71 and 85%, respectively, at 550 nm), which can be attributed to their anisotropic light scattering behavior due to the natural alignment of the holo-fibers.

Table 2 summarizes the main optical outcomes related to the different TW systems discussed in the section.

### 3.3. Poplar Transparent Wood

Tang and co-workers [86] fabricated a highly flexible, TW film with a thickness of 150 μm from poplar veneers through a chemical delignification process followed by polymer infiltration and subsequent UV-curing. Delignification was performed by immersing the veneers in a 4 wt.% NaClO_2_ solution (buffered with glacial acetic acid at pH = 4–5) at boiling temperature for 3 h. The delignified veneers were then vacuum-infiltrated with poly(ethylene glycol) diacrylate (average MW: 700 g/mol) embedding 1 wt.% of the photoinitiator. The impregnated veneers were finally UV-cured (radiation intensity on the wood surface: 120 W/cm^2^) for up to 40 s. The resulting TW film showed a high optical transmittance (beyond 90%) within the visible wavelength range and noticeable haze (exceeding 70%), which was attributed to residual interfacial scattering between the wood cell walls and the polymer.

Bisht et al. fabricated a TW from 2 mm thick longitudinal poplar veneers using a two-step lignin modification and bleaching process, followed by the infiltration of an epoxy resin [87]. The bleaching solution was kept at 70 °C and consisted of 3 wt.% sodium hydroxide, 0.1 wt.% ethylenediamine tetraacetic acid disodium salt, and 0.1 wt.% magnesium sulfate. Then, hydrogen peroxide (25 vol.%) was added, and the reaction continued for approximately 3 h until the wood samples turned completely white. Next, the bleached veneers were vacuum-infiltrated with an epoxy-hardener mixture and cured at ambient temperature for 24 h. The resulting TW had an optical transmittance of about 84% at 550 nm and a haze value of around 92%: the latter was attributed to light scattering phenomena occurring within the wood-polymer microstructure.

Poplar slices (0.65 mm thick) were employed by Liu and co-workers to produce a photochromic TW. For this purpose, delignification was performed using 1 wt.% NaClO_2_ solution, working at pH = 4.6 at 80 °C for 4 h [88]. Then, the delignified wood was vacuum-infiltrated with pre-polymerized methyl methacrylate embedding different amounts of dithienylcyclopentene as photochromic dye (in between 0.02 and 0.1 wt.%). The polymerization was carried out at 75 °C for 5 h. The so-obtained TW showed high optical transparency, UV/visible switchable coloration, and UV-shielding, with optical transmittance above 80% in the visible wavelength range.

Zou et al. [89] produced superthin TW films (90 μm thick) from poplar veneer slices cut perpendicularly to the tree’s growth. First, the slices underwent delignification in a 4 wt.% NaClO_2_ solution at 100 °C for 1.5 h, maintaining a pH between 4 and 5. The delignified material was then infiltrated with a flexible epoxy resin containing CdSe/ZnS quantum dots at various concentrations (namely, 0.017, 0.036, and 0.078 wt.%). Finally, the infiltrated wood was cured at room temperature for 1 day. Due to the excellent match between the RIs of the epoxy system and delignified wood, the resulting TW films exhibited optical transmittance of up to 90% in the visible wavelength range. In addition, these films retained uniform luminescence under environmental exposure.

Chen and co-workers [90] delignified 0.5 mm thick poplar wood slices in a 1 wt.% sodium chlorite solution buffered at pH = 4.6, working at 80 °C for up to 6 h. To create a hydrochromic TW (HTW), delignified slices were vacuum-infiltrated with a pre-polymerized PMMA solution containing 0.036 wt.% oxazolidine-based hydrochromic molecules. Then, the slices underwent polymerization at 75 °C for 4 h. The resulting HTW exhibited high optical transmittance (over 80%) in the visible wavelength range and humidity-dependent color changes caused by reversible oxazine ring opening or closing.

Wu et al. [91] succeeded in providing 2 mm thick, poplar-derived TW with multifunctional features, namely high optical transmittance (beyond 93%), self-cleaning, and superhydrophobicity (with water contact angle values as high as 163.5°). To achieve this, poplar veneers underwent delignification in a 5 wt.% NaClO_2_ solution buffered with glacial acetic acid and operated at 85 °C for 6 h. Then, the delignified wood was vacuum-infiltrated with a 1:1 mixture of UV-curable urethane acrylate and epoxy acrylate, and UV-cured for 200 s. Finally, the surface of the TW was functionalized with SiO_2_ nanoparticles modified by vinyltriethoxysilane to provide hydrophobic properties. These properties suggested the suitability of the obtained TW for photovoltaic applications.

Table 3 summarizes the main optical outcomes related to the different TW systems discussed in the section.

### 3.4. Basswood Transparent Wood

Jiang et al. [92] fabricated a hemicellulose-rich TW fabricated using basswood (Tilia) slices (0.37 mm thick). First, the native wood slices were delignified with a 4 wt.% peracetic acid solution at pH 4.8 (adjusted with NaOH) and 80 °C. Then, the delignified wood was vacuum-infiltrated with a pre-polymerized methyl methacrylate solution for 3 h and polymerized at 75 °C for 4 h. The final thickness of the TW slices was approximately 0.5 mm. The TW obtained showed high optical transmittance (about 85%) and medium haze (about 72%) at 550 nm, thanks to the microstructural integrity of the hemicellulose network. Additionally, the TW slices exhibited stable optical behavior even after 7 days of exposure to sunlight.

Liu and co-workers [93] produced low-haze, water-resistant TW films from basswood slices. To this end, the native wood was delignified using a 1 wt.% NaClO_2_ solution buffered at pH 4.6 with acetic acid, operating at 80 °C for 12 h. The resulting material was compressed at room temperature with 5 MPa of pressure applied for 3 h to obtain densified TW films with a thickness of about 0.094 mm. Then, the surface was brushed with an epoxy resin system and cured at 60 °C in an oven. At 550 nm, the epoxy-coated TW films showed high transmittance (approximately 80%) and low haze (approximately 43%) due to limited interfacial scattering. Additionally, the final material exhibited similar optical behavior in agitated water.

**Figure 14:**
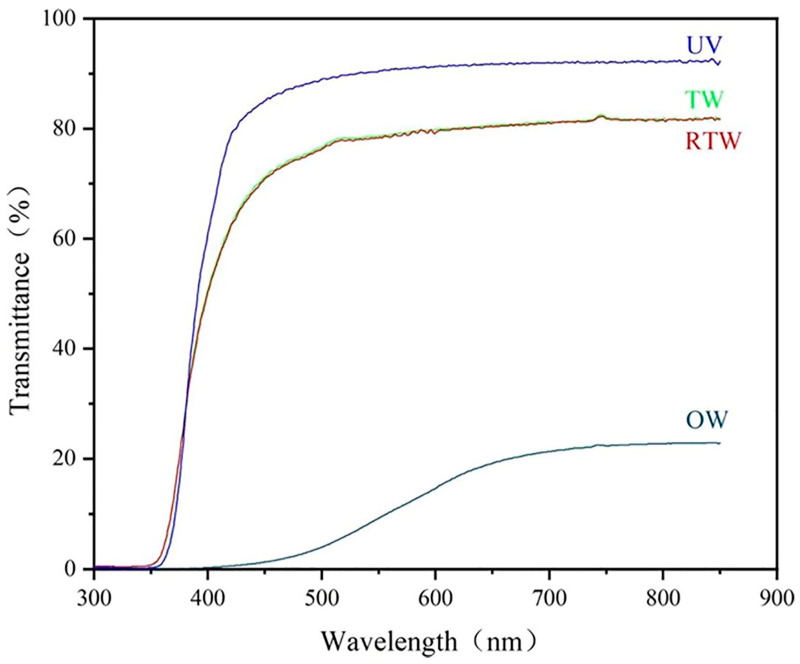
Optical transmittance of original wood (OW), transparent wood (TW), reactive dyed transparent wood (RTW), and UV-cured resin (UV). Reprinted from [94] under CC-BY-NC-ND-4.0 License.

Zhao et al. [94] succeeded in obtaining colored, transparent basswood slices. They delignified 0.5 mm thick veneers using a 3.6 wt.% NaClO_2_ solution that was buffered to a pH of 4.6 with acetic acid. The solution was operated at 85 °C for 2 h. Then, the delignified wood was dyed in an aqueous solution of reactive red X-3B for 2 h using sodium sulfate and sodium carbonate as auxiliaries. After washing, the dyed, delignified wood was vacuum-infiltrated with a commercially available UV-curable resin mixture containing polyurethane acrylate, polyester acrylate, epoxy acrylate, and acrylamide morpholine for 24 h. Then, it was UV-cured at room temperature. As shown in Figure 14, the resulting red-dyed TW showed an optical transmittance of around 81% in the visible wavelength range. This value was comparable to the transmittance of the undyed TW, indicating that the dyeing process did not compromise transparency. Additionally, the dyed TW exhibited enhanced color stability, demonstrating smaller color differences after UV exposure compared to the undyed TW, which confirmed enhanced yellowing resistance.

Table 4 summarizes the main optical outcomes related to the different TW systems discussed in the section.

### 3.5. Fir Transparent Wood

A TW composite with retained wood color and texture was fabricated from Chinese fir (*Cunninghamia konishii*) through partial delignification followed by surface modification using a silane coupling agent and epoxy resin infiltration [95]. The wood veneers (thickness: 0.45 mm) were pretreated in a 1 wt.% NaClO_2_ solution (buffered at pH 4.6 with glacial acetic acid) at 90 °C for 1.5 h to partially remove the lignin and produce a semi-transparent wood template. This template was then impregnated with ethanolic solutions of silane coupling agent (3–7 wt.%) for different times (20–60 min) at various temperatures (15–40 °C) to optimize interfacial compatibility. An orthogonal experimental design method was employed for this purpose. After modification, the samples were vacuum-infiltrated with an epoxy resin system and subsequently cured at room temperature for up to 18 h. Using the optimized conditions (5 wt.% silane coupling agent, 20 min reaction time, and 25 °C reaction temperature), the TW reached an optical transmittance of over 87% at 800 nm while maintaining a haze of less than 36% and preserving the natural color and grain of the native wood. These results highlighted the suitability of the material for decorative structural applications.

In a further research effort, the same group [96] designed an antibacterial and aesthetically pleasing TW by combining partial delignification and chitosan-epoxy resin infiltration. Specifically, 1 mm thick fir veneers were immersed in a 1 wt.% NaClO_2_ solution (pH 4.7) at 90 °C for 100 min. After washing, the templates were infiltrated with an epoxy resin system adjusted to a pH of less than 6.5 and containing different amounts of chitosan (from 0.5 to 4 wt.%) under vacuum. The samples were then cured for 24 h at room temperature. The TW modified with chitosan exhibited adjustable optical properties. The transmittance at 550 nm was approximately 74%, and the haze increased with the amount of chitosan, reaching approximately 87% in TW with the highest chitosan content. The composites also showed strong UV-shielding below 400 nm. Finally, incorporating chitosan was found to enhance uniformity and optical stability while preserving the warm color and natural texture of the original wood.

Pursuing this research, an optically reversible, phase-change thermal storage TW, employing dodecanol as the functional phase-change additive, was designed and produced [97]. To achieve this, fir wood slices (thickness: 1 mm) were first crosslinked using 1,4-butanediol diglycidyl ether. Then, two delignification methods were performed: delignification in a 2 wt.% NaClO_2_ solution (pH = 4.6, buffered with glacial acetic acid) and in a 1:1 H_2_O_2_/acetic acid mixture, both operating at 80 °C. Next, the delignified samples were vacuum-infiltrated with epoxy resin containing different amounts of dodecanol (from 50 to 80 wt.%) at 60 °C for 1 h and then cured at 75 °C for 5 h. The sample infiltrated with epoxy resin containing 70 wt.% dodecanol exhibited high optical transmittance (around 78%) and low haze (33%) at 800 nm above the phase transition temperature (around 26 °C). Below this temperature, the optical transmittance decreased to approximately 48%, and the haze increased to approximately 86%, demonstrating remarkable optical reversibility. Finally, it is worth noting that the TW maintained stable properties after 100 freeze–thaw cycles and effectively delayed indoor temperature rise when used for energy-saving glazing.

Table 5 summarizes the main optical outcomes related to the different TW systems discussed in the section.

### 3.6. Maple Transparent Wood

Cheng and co-workers [98] developed a transparent, wood-based triboelectric nanogenerator from maple by combining delignification with a NaClO_2_ solution and the infiltration of a UV-curable resin. To achieve this, 0.4 mm thick veneers were delignified in a 3.8 wt.% NaClO_2_ solution buffered at pH 4.6 with glacial acetic acid at 85 °C for up to 3 h. The delignified wood templates were then vacuum-infiltrated with a UV-curable resin for 24 h and then cured with UV radiation for 5 min. The TW exhibited an optical transmittance of around 89% and a haze of about 52% at 800 nm, making it suitable for use in optoelectronic or energy harvesting devices.

The same group pursued this research by further developing a flexible triboelectric nanogenerator based on TW through controlled delignification and infiltration with an epoxy resin system [99]. In particular, 0.5 mm thick veneers delignified using the previously described method were soaked in ethanol for 24 h. Then, they were vacuum-infiltrated with an epoxy resin for 3 h and finally cured between glass plate films. The optical transmittance exceeded 89%, and the haze was around 57%. Notably, all the TW samples maintained the natural amber texture of maple. Additionally, the triboelectric nanogenerator incorporating the TW produced an open-circuit voltage of 127 V and demonstrated excellent durability over 10,000 cycles. This confirmed its potential for use in self-powered sensors and transparent energy harvesting devices.

Xu et al. [100] fabricated a photoluminescent TW with UV-shielding capability by employing Canadian white maple as the base material through delignification, carbon quantum dot incorporation, and epoxy resin infiltration. The delignification step involved soaking 0.5 mm thick veneers in a 3.7 wt.% NaClO_2_ solution buffered at pH 4.6 with glacial acetic acid at 80 °C for 2 h. Then, carbon quantum dots synthesized from citric acid and urea under microwave irradiation were dispersed in 0.1‰ ethanol and allowed to infiltrate the delignified wood template for 48 h. The samples were then vacuum-infiltrated with epoxy resin for 3 h and finally cured at room temperature for 24 h. The resulting photoluminescent TW achieved an optical transmittance of approximately 91% in the visible wavelength range and a haze of over 70%. The carbon quantum dots imparted strong UV absorption (within 320 and 400 nm) and yellow fluorescence under 395 nm excitation to the TW without affecting its mechanical integrity.

Table 6 summarizes the main optical outcomes related to the different TW systems discussed in the section.

### 3.7. Bamboo Transparent Wood

Although bamboo is technically a type of grass and not wood [30], several recent papers have investigated its optical performance. The main findings are discussed below.

Wang and co-workers [101] exploited a bleaching process involving the treatment of 1.5 mm thick Moso bamboo slices in an alkaline solution containing 4 wt.% H_2_O_2_, 3 wt.% NaOH, 3 wt.% Na_2_SiO_3_, 0.1 wt.% MgSO_4_, and 0.1 wt.% diethylenetriaminepentaacetic acid at 70 °C until complete whitening was achieved. This process retained approximately 78% of the lignin content, thereby preserving the integrity of the bamboo cell walls. The bleached bamboo was vacuum-infiltrated with an epoxy resin and cured at room temperature for the required time. The transparent bamboo exhibited an optical transmittance of 87% and a haze of 90% within the visible wavelength range while maintaining a high tensile strength of around 118 MPa and a low thermal conductivity of approximately 0.33 W/(mK).

To overcome thickness and anisotropy limitations, Wang et al. [102] fabricated a multilayer transparent bamboo through lamination and epoxy infiltration. First, 0.3 mm thick Moso bamboo veneers were delignified in a 3.5 wt.% NaClO_2_ solution buffered at pH 4.6 with glacial acetic acid at 80–90 °C for up to 3 h. The resulting templates were vacuum-infiltrated with an epoxy resin system, laminated in parallel or cross orientations (piling up from 3 to seven layers), and finally cured at 25 °C for 12 h. The optimal configuration, with five parallel layers and a total thickness of 1.5 mm, produced an optical transmittance of about 82% and a haze of 72% within the visible wavelength range. In contrast, cross-laminated structures offered enhanced dimensional stability and UV resistance. The ability to tune the optical transmittance depending on the lamination sequence and the number of layers accounted for a high flexibility in designing decorative and structural transparent panels.

Wang and co-workers [103] exploited an alkali pretreatment-crosslinking-delignification strategy to produce large, flexible, transparent bamboo samples. To achieve this, bamboo veneers (1.0 mm thick) were first pretreated in 1 wt.% NaOH at 80 °C. Then, they were crosslinked with 1,2,3-propanetriol diglycidyl ether for 6 h. Finally, they were delignified with 1 wt.% NaClO_2_ buffered at pH 4.6 with glacial acetic acid at 80 °C. The delignified templates were then infiltrated with a flexible epoxy resin system and cured at 60 °C for the required time. The 1 mm thick transparent bamboo samples exhibited 80% optical transmittance, 72% haze, good tensile strength (around 78 MPa), and low thermal conductivity (0.35 W/(mK)). The proposed method can produce transparent bamboo sheets up to 15 cm wide, suggesting potential for industrial-scale fabrication.

Zhang et al. [104] produced large-sized photochromic transparent bamboo by combining crosslinking, delignification, and infiltration with an epoxy resin system containing photochromic pigments. To achieve this, bamboo veneer slices (100 × 100 × 0.9 mm^3^) were crosslinked with 1,2,3-propanetriol diglycidyl ether in an alkaline ethanol solution, then delignified in a 1 wt.% NaClO_2_ solution at 80 °C. Then, the delignified bamboo template was vacuum-infiltrated with an epoxy resin system containing 0.5 wt.% photochromic microcapsules and cured at 60 °C for 24 h. The resulting material exhibited an optical transmittance of 78% and a haze of over 70%, as well as reversible, UV-responsive coloration. Additionally, its tensile strength and thermal conductivity were approximately 85 MPa and 0.36 W/(mK), respectively. The large size of the transparent, photochromic bamboo, combined with its unique properties (light modulation, energy efficiency, and mechanical strength), made it a potential candidate for smart window applications.

Zhang and co-workers [105] used the gradient structure of bamboo to design and produce a flexible bamboo composite with adjustable optical transmittance and fiber texture. To achieve this, they delignified veneers from different layers of the bamboo wall (from outer to inner) in a 3 wt.% NaClO_2_ solution buffered at pH 4.6 with glacial acetic acid, operating at 60 °C for 12 h. They then infiltrated the veneers with an epoxy resin system. Single- and multi-layer transparent bamboo samples (0.6–1.5 mm) were obtained through parallel or cross lamination; the subsequent curing was performed at room temperature for 12 h. The optical transmittance of the resulting laminates depended on fiber volume content. More specifically, it was approximately 45% for low-density veneers and 10–12% for high-density veneers. Haze increased with increasing the fiber content.

Table 7 summarizes the main optical outcomes related to the different transparent bamboo systems discussed in the section.

**Figure 15:**
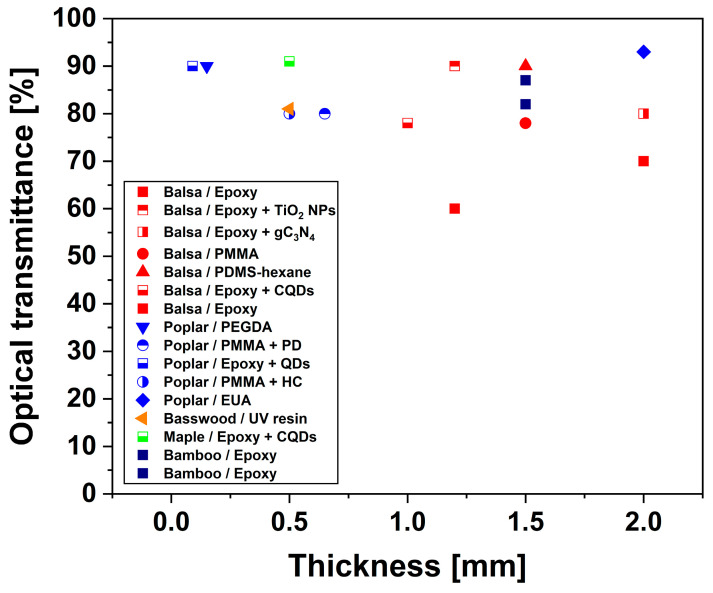
Scatter diagram of optical transmittance vs. thickness for different TWs. The acronyms are defined in the abbreviation list.

**Figure 16:**
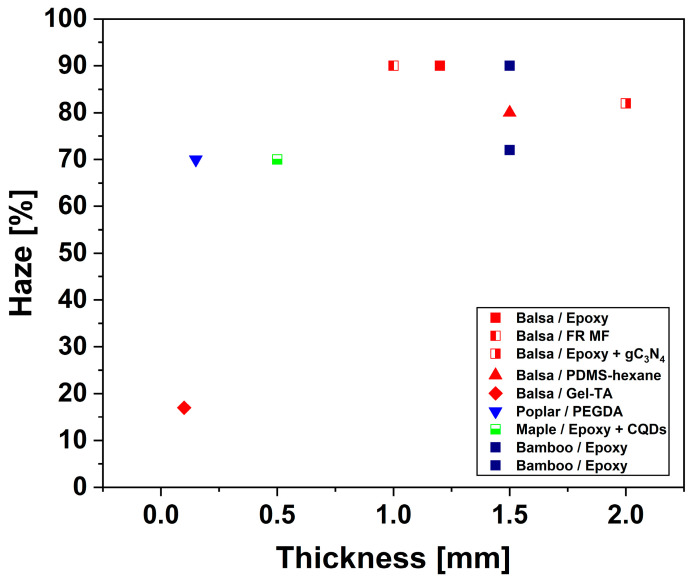
Scatter diagram of haze vs. thickness for different TWs. The acronyms are defined in the abbreviation list.

Figure 15 and Figure 16 provide a graphical representation of the effect of thickness on the optical transmittance and haze of TWs, respectively. Further, an application-oriented discussion is reported in Section 3.8.

### 3.8. Application-Oriented Discussion

TW has a variety of uses thanks to its high optical transmittance, controllable haze, and tunable RI. These properties make it suitable for a broad range of optical and structural applications. However, specific optical requirements differ according to intended function, such as architectural glazing, photovoltaic encapsulation, or light-diffusive components, necessitating a contextual evaluation of current systems against these criteria.

For architectural applications (building glazing and curtain wall systems), the desired optical parameters include total transmittance of 80–90% at 550 nm, haze values below 60%, a color difference (ΔE*) below 3, and high resistance to yellowing and photodegradation. Among the reported systems, balsa-PMMA, birch-PMMA, and fir-silane-modified epoxy composites meet these benchmarks by combining high *T* (85–94%) with moderate haze (30–55%) and stable colorimetric properties [93,95]. Furthermore, lignin-retaining and surface-modified systems demonstrate superior weatherability: in fact, they maintain transparency and chromatic stability after prolonged UV exposure. This is essential for façade and daylighting applications.

In photovoltaic applications, the optimal configuration for solar energy systems favors high haze (>80%), high transmittance (>85%), and strong UV shielding to enhance light trapping and extend the lifetime of the device. TWs made from balsa or poplar that are infiltrated with epoxy or phosphate ester-polyethylene glycol often have haze levels above 90% and UV-blocking efficiency of up to 100% [65]. These TWs are particularly promising for this application. The combination of forward scattering and UV absorption enables an increased photon path length and improved photovoltaic efficiency while maintaining mechanical integrity and environmental sustainability.

For interior lighting and optical diffusion panels, uniform light scattering is the primary requirement rather than clarity. TWs with high haze (between 80 and 95%) and balanced transmittance (from 70 to 85%) provide soft, homogeneous illumination without glare, making them ideal for diffusers and privacy glazing. Balsa-epoxy, balsa-PLIMA, and bamboo-epoxy laminates are systems that demonstrate the desired combination of optical diffusion and mechanical robustness [76,94]. The uniform haze distribution across these materials ensures consistent brightness and reduced optical anisotropy, which are attributes advantageous in luminaire design and architectural interiors.

In summary, TW systems can be functionally classified by their optical performance. In particular, low-haze, high-transmittance TWs (e.g., PMMA- or silane-based systems) can be used for structural glazing and curtain walls, while high-haze, UV-shielding TWs (e.g., epoxy- or PEG-based systems) may find application in the photovoltaic sector. High-haze, uniformly scattering TWs (e.g., PLIMA-, epoxy-, or bamboo-based composites) are employable for lighting diffusion and privacy applications [106,107].

## 4. Research Gaps and Future Directions in TW

Despite significant advances in TW technology have been made, critical challenges remain, limiting the material’s scalability and optical optimization. These challenges can be summarized as follows.

*Thickness-dependent transparency*: although numerous studies have demonstrated high transmittance in thin TW films (less than 1 mm thick), a systematic understanding of the attenuation mechanisms in thicker samples is lacking. The exponential decrease in light transmission with increasing thickness is often attributed to multiple scattering, anisotropic fiber alignment, and inhomogeneous monomer infiltration. However, quantitative models correlating optical path length, microstructural anisotropy, and scattering coefficients are scarce. Developing predictive, thickness-dependent optical models supported by Monte Carlo simulations or radiative transfer analyses would enable the more rational design of centimeter scale TWs with consistent optical clarity.

*Haze management and reduction strategies*: haze remains an intrinsic limitation of high-clarity TW because forward scattering caused by residual RI mismatches and nanoscale air voids persists even in optimized systems. Current approaches, such as polymer selection, hot compression, and surface densification, offer only partial mitigation and are not universal. Future work should focus on: (i) engineering RI-tunable polymer systems that can dynamically match the RI of the delignified/bleached wood template under varying humidity and temperature conditions; (ii) optimizing monomer infiltration kinetics through controlled viscosity and pressure cycling; (iii) employing interface coupling agents or atomic-layer coatings to minimize interfacial voids and scattering centers. Additionally, nanoscale topographical smoothing and hybrid nanofiller incorporation may reduce diffuse scattering without compromising the overall mechanical strength of the final material.

*Standardization and reproducibility*: a major obstacle to cross-comparison across studies is the absence of standardized measurement protocols for optical transmittance and haze. In fact, using different integrating sphere geometries, aperture angles, and spectral bandwidths may lead to inconsistent data. Therefore, establishing international testing standards tailored for such anisotropic materials as TW is essential for reproducible benchmarking.

*Scale-up and structural uniformity*: the optical quality of TW often deteriorates when scaling up to large-area or multi-millimeter specimens due to incomplete delignification, monomer infiltration gradients, and microcracks induced by residual stresses. Future research should focus on scalable flow-assisted infiltration, real-time curing monitoring, and gradient-free delignification technologies to achieve uniformity across macroscopic dimensions.

*Multifunctional and sustainable design*: although multifunctionality has been demonstrated (e.g., self-cleaning, photochromism, and phase-change behavior), simultaneously optimizing optical, mechanical, and environmental performance remains challenging. Prioritizing bio-based polymers with tunable RI and recyclability potential will ensure circular manufacturing. Integrating eco-friendly, lignin-retaining, or lignin-modifying chemistries may enhance optical uniformity while maintaining sustainability.

In summary, advancing TW from a laboratory material to an industrial optical composite requires an integrated approach that combines thickness-dependent optical modeling, interface nanoengineering, and standardized optical evaluation. This approach must be supported by sustainable polymer chemistry and process scale-up strategies. Therefore, addressing these research gaps is crucial for achieving glass-like clarity in thick, large-area TW structures with stable and reproducible optical performance.

## 5. Conclusions and Perspectives

TW is emerging as an interesting material that preserves the cellular microstructure of wood while removing the lignin or bleaching the light-absorbing constituents (i.e., lignin’s chromophores) and infiltrating the resulting template with a transparent polymer system showing a comparable RI. The resulting material exhibits high optical transmittance (often beyond 70–85% for millimeter-scale thicknesses) coupled with substantial forward scattering (high haze), enabling diffusive daylighting. However, achieving glass-like clarity at comparable thicknesses while maintaining sustainability, dimensional stability, and scalability remains challenging.

In fact, despite impressive gains in optical transmittance, the optical performance of state-of-the-art TW is still limited by intrinsic scattering, anisotropy, and processing-induced defects.

The overall optical performance of TWs is typically summarized by total transmittance and haze. The latter quantifies the fraction of transmitted light that is scattered forward outside a narrow acceptance cone. TW typically exhibits high transmittance and high haze due to RI discontinuities at numerous interfaces and anisotropic microstructures that bias scattering along the wood growth direction. Reported values of around 80–85% and 60–75% for optical transmittance and haze, respectively, for 1 mm thick TW samples are representative benchmarks for methyl methacrylate- or epoxy-infiltrated delignified wood templates. While these metrics are ideal for glare-free illumination, applications requiring view-through transparency, such as display covers or windowpanes, necessitate high imaging clarity (i.e., low haze). It is important to note that both transmittance and haze worsen as thickness increases, and the rate of worsening depends on the density of interfaces, index mismatch, and defect population.

The dominant source of haze in TW is forward scattering caused by a mismatch in RIs between the cell wall phase (cellulose/hemicellulose) and the infiltrated polymer. Even modest differences in RI values produce cumulative scattering across billions of interfaces per cubic centimeter. Localized debonding at the wood-polymer interface and the presence of residual voids exacerbate the mismatch by introducing air gaps with a low RI that create strong scattering centers.

The optical transmittance significantly decreases with thickness when multiple scattering and absorption are present. Meanwhile, haze accumulates with optical path length through repeated small-angle scattering events. Consequently, achieving high clarity in multi-millimeter or centimeter-scale TW slices is much more difficult than in thinner counterparts.

Additionally, surface roughness, microcracks resulting from drying or thermal expansion mismatch, and processing defects (e.g., incomplete monomer infiltration and the presence of entrapped microbubbles) contribute to additional scattering. Poor wetting of the delignified wood template by the monomer(s) used for infiltration, insufficient vacuum/pressure cycling, and polymerization shrinkage can also leave nanoscale voids and debonded regions.

Another important limiting factor of TW is its long-term stability under environmental stressors. This remains a critical bottleneck for its practical outdoor implementation. Few studies have quantitatively examined how transmittance, haze, and chromaticity evolve under extended UV exposure, damp-heat conditions, or thermal cycling. For example, Bisht et al. [108] observed a gradual decline in transmittance and an increase in haze after prolonged natural weathering. This was accompanied by noticeable yellowing due to the photodegradation of the polymer matrix and residual lignin. Zhao and co-workers [94] investigated the chromatic stability of reactive-dyed transparent basswood under UV exposure. They demonstrated improved stability due to dye-assisted UV absorption, showing only a minor chromaticity change and a negligible transmittance loss after 120 h of accelerated irradiation. Wu et al. [82] investigated UV-shielded TW with an interface modification and found that it had stable optical transmittance (>85%) and a chromaticity change lower than 2 after cyclic UV and moisture exposure. This confirmed the effectiveness of UV-blocking layers in suppressing color drift. Zhou and co-workers [97] provided more precise measurements of the phase-change thermal performance, revealing slight variations in transmittance (±3%) and a chromaticity change below 2 after 100 freeze–thaw cycles. These results confirmed the reversibility of both the chromatic and structural properties. Barbosa et al. [39] demonstrated that extended exposure to sunlight led to polymer oxidation and interface debonding in TW, ultimately altering its light scattering behavior. Despite the incorporation of UV absorbers or hydrophobic coatings, the cumulative effects of photo-oxidation, moisture diffusion, and thermal stress on the optical and mechanical integrity of TW remain unclear.

Therefore, systematic accelerated aging experiments that include combined UV irradiation, humidity/heat cycling, and freeze–thaw testing are urgently needed to simulate real service conditions and track the concurrent evolution of optical parameters. Correlating these tests with natural weathering data would allow us to predict lifetime performance and provide design guidelines for outdoor architectural, photovoltaic, and daylighting applications. Future studies should focus on establishing standardized protocols for aging-optical coupling analysis involving in situ spectroscopic and colorimetric monitoring to elucidate degradation kinetics and failure mechanisms. Thus, achieving durable optical performance requires coupling optical optimization with environmental stability solutions through the use of UV-resistant, hydrophobic, and thermally stable monomer systems, as well as chemically bonded wood-polymer interfaces that can maintain transmittance and low chromatic drift under prolonged outdoor exposure.

Another current issue is the lack of reproducibility when making TW across different species and growth directions. There is also a lack of standardized measurement protocols, such as integrating sphere geometries and haze apertures.

Additionally, scaling up to larger wood slices introduces gradients in reagent diffusion during delignification and in monomer infiltration kinetics. These gradients drive spatial variation in optical properties.

Several strategies have been employed to address these issues, and further implementation is necessary.

First, it is crucial to select specific monomers that will infiltrate the delignified wood templates because the RI of the corresponding polymers should closely match that of the delignified cell walls, which is usually around 1.5, depending on the moisture content and orientation. This reduces the RI mismatch. Copolymerization or blending can increase the polymer’s RI without excessive absorption, thus maintaining acceptable optical transparency.

Then, engineering the interface between the wood cell walls and the infiltrated monomers could be an effective strategy. In this context, using suitable coupling agents, such as silanes, or utilizing surface activation techniques, such as plasma activation [109] or mild/controlled oxidation [60,66], can promote chemical bonding between hydroxyl-rich cell walls and polymer matrices and suppress debond gaps.

Furthermore, the presence of microvoids, which act as scattering centers for incident light, can be significantly reduced by optimizing infiltration vacuum/pressure cycles, degassing infiltration baths, and performing slow curing processes to prevent shrinkage. In this context, using reactive diluents to lower viscosity and ensure lumen and nanoscale pore filling before monomer gelation is a promising approach.

Recently, some lignin-retaining methods have been explored, demonstrating their ability to provide TW with high optical and mechanical quality. The selective removal of chromophoric moieties while preserving much of the lignin network limits cell wall damage and retains the material’s mechanical integrity. In combination with good RI matching, this strategy can mitigate scattering phenomena caused by over-delignification while enhancing dimensional stability and providing high optical transmittance.

Similarly, hot compression and densification methods increase the volume fraction of cellulose and favor the collapse of large lumina that behave as low-RI cavities. This limits the occurrence of large-angle scattering phenomena. These approaches are important when thin, transparent TW slices are required.

It is also essential to highlight the key role of the TW surface in providing multifunctional features to the material. In this context, some research is being devoted to designing TW surfaces that exhibit high hydrophobicity, which limits fluctuations in the RI due to moisture absorption. These surfaces may also provide UV-shielding and protection, reducing yellowing and stabilizing optical features. Additionally, modifying the infiltrated monomers by incorporating different additives (e.g., phase change materials, photochromic nanofillers, luminescent nanofillers, and flame retardants) surely contributes to the multifunctionality of the obtained TW without substantially altering its optical properties.

Finally, the well-known and up-to-date circular economy concept addresses the use of bio-sourced monomers with high RIs and cellulose-compatible networks to retain the sustainability edge. This strategy is promising, though the portfolio of monomers with the required characteristics is still limited.

In the coming years, achieving glass-like clarity in centimeter-scale TW will likely require further research addressing the precise tuning and engineering of the RI of infiltrated polymers, the development of chemically robust interfaces with a near-zero void fraction; the management of anisotropy and thickness effects; and the establishment of durable, UV- and moisture-stable chemistries.

Given the rapid pace of innovation in RI-tunable polymers, lignin-preserving chemistries, and process analytics, it is plausible that significant reductions in haze will be achieved at application-relevant thicknesses in the near term while retaining wood’s unique properties, such as its mechanical behavior and sustainability advantages.

## Figures and Tables

**Table 1:** Main optical properties of transparent balsa woods.

Delignification Method	Infiltration Process and Polymerization/Curing	TW Thickness (mm)	Main Optical Outcomes	Ref.
Oxidation of lignin chromophores with H_2_O_2_ and UV radiation	Flame retarded melamine formaldehyde resin	2	- High optical transmittance (up to 95%) at 600 nm - High haze level (around 96%)	[59]
Treatment with acidic NaClO_2_ solution	Vacuum impregnation with a UV-curable resin and possible compression step	0.7	- 81% optical transmittance and 67% haze at 550 nm after compression - 47% optical transmittance and 95% haze at 550 nm without compression	[41]
Treatment with acidic NaClO_2_ solution, subsequent TEMPO-mediated oxidation and final densification	None	2	- Optical transmittance and haze of around 75%	[60]
Treatment with acidic NaClO_2_ solution	Infiltration with an epoxy vitrimer and curing	2	- Optical transmittance of 60% at 600 nm - High haze level (around 95%) at 600 nm	[61]
Treatment with acidic NaClO_2_ solution	Infiltration of an epoxy resin containing TiO_2_ nanoparticles and curing	1.2	- Limited optical transmittance (around 60%) in the visible region - Increased transmittance (up to 90%) in the presence of TiO_2_ nanoparticles - Haze (around 90%) unaffected by the nanofiller	[62]
Bleaching treatment at 70 °C or treatment with acidic NaClO_2_ solution at 80 °C	Infiltration with a flame retarded melamine formaldehyde resin and curing at 150 °C for 15 min	1	- About 65% optical transmittance at 600 nm for the bleached TW - About 60% optical transmittance at 600 nm for the delignified TW - High haze level (around 90%) within the visible region for both bleached and delignified TWs	[63]
Treatment with acidic NaClO_2_ solution at 80 °C	Infiltration with a vitrimeric mixture and curing up to 60 °C	2	- 90% optical transmittance and haze at 800 nm for TW cut in the transverse direction - 71 and 72% of optical transmittance and haze, respectively, at 800 nm for longitudinally cut TW	[64]
Treatment with H_2_O_2_ for 48 h	Infiltration with a phosphate ester-polyethylene glycol	-	- 93% optical transmittance in the entire visible region - 98% haze in the entire visible region	[65]
Treatment with acidic NaClO_2_ solution at 80 °C and subsequent TEMPO-mediated oxidation	Infiltration with alkali lignin	1	- 48% optical transmittance at 800 nm	[66]
Treatment with acidic NaClO_2_ solution at 80 °C for 48 h	Infiltration of poly(vinyl alcohol) incorporating MXene nanosheets	1	- Decreased optical transmittance and increased haze with increasing amounts of MXene nanosheets - Progressive color darkening	[67]
Treatment with acidic NaClO_2_ solution at 70 °C for 24 h	Infiltration with a bisphenol A diglycidyl ether-based epoxy resin and curing	2.5	- 67 and 73% of optical transmittance and haze, respectively, at 750 nm	[68]
Treatment with acidic NaClO_2_ solution at 80 °C	Infiltration of polythiourethane vitrimers	1 or 2	- 87 and 82% of optical transmittance for 1 and 2 mm thick TW specimens, respectively, - 73 and 89% haze for 1 and 2 mm thick TW specimens, respectively	[69]
Treatment with acidic NaClO_2_ solution at 90 °C and subsequent treatment with H_2_O_2_ at 90 °C for 1 h	Infiltration with a glucose/phenol-derived resin under vacuum at room temperature, and final curing	0.8	- 46% optical transmittance at 800 nm	[70]
UV exposure of the native wood immersed in an aqueous solution of H_2_O_2_ and ammonia	Infiltration with an epoxy system under vacuum for 3 h and final curing at room temperature for 1 day	1	- Increased optical transmittance with infiltration time - Gradual decrease in haze with infiltration time	[71]
Solar-assisted bleaching or steam bleaching or NaOH delignification	Vacuum infiltration of a poly(vinyl alcohol)/propylene glycol mixture embedding lignin nanoparticles and final drying	1	- 80% optical transmittance achieved by NaOH delignified TW between 600 and 800 nm - Lower optical transmittance values for solar-assisted (about 60%) and steam bleached (65%) TWs	[72]
Treatment with acidic NaClO_2_ solution at the boiling point for 2 h, subsequent oxidation in NaIO_4_ solution at 50 °C for 4 h, densification at 60 °C for 48 h, and final lamination in a three-ply structure	None	3	- 85% optical transmittance in the visible region - Very low haze (about 20%) in the visible region	[73]
Treatment in NaOH solution at 90 °C for 2 h	Infiltration of an epoxy system incorporating graphite carbon nitride and curing at room temperature for 72 h	2	- Over 80% optical transmittance between 500 and 800 nm - Very high haze values (about 82%) in the same wavelength range	[74]
Brushing with H_2_O_2_ and NaOH and subsequent exposure to UV radiation for 20 min	Vacuum infiltration with pre-polymerized methyl methacrylate for 24 h and polymerization at 70 °C for 4 h	1.5	- In the cold state, 78% optical transmittance in the visible region - In the hot state, 25% optical transmittance in the visible region	[75]
UV exposure of the native wood immersed in an aqueous solution of H_2_O_2_ and ammonia	Dipping in polydimethylsiloxane-hexane solution for 10 min and curing at 80 °C for up to 4 h	1.5	- 90% optical transmittance and 80% haze within 400 and 700 nm	[76]
Treatment with acidic NaClO_2_ solution at 85 °C	Preparation of a three-layer TW structure, where the core was vacuum-infiltrated with polyethylene glycol-silica for 2 h, while the outer layers were infiltrated with methyl methacrylate and thermally polymerized	-	- 45% optical transmittance and 76% haze at 800 nm	[77]
Treatment with acidic NaClO_2_ solution at 80 °C for 6 h	Vacuum infiltration with bisphenol A diglycidyl ether-based epoxy resin incorporating carbon quantum dots and final curing	1	- 78% optical transmittance in the visible region - 79 and 78% UV-B and UV-A radiation blocking, respectively	[78]
Treatment with a mixture of glacial acetic acid and H_2_O_2_ at 80 °C for 6 h	Vacuum infiltration with an aqueous solution of an ionic liquid, subsequent removal of the latter, and drying under vacuum at 60 °C for 4 h for densification	0.15	- 70% optical transmittance and 95% haze	[79]
Treatment in acidic NaClO_2_ solution at the boiling point for 2 h, removal of hemicellulose with NaOH solution at 40 °C for 2 h, and final oxidation in NaIO_4_ solution at 50 °C for 4 h	Densification, subsequent grafting with a gelatin solution at 60 °C for 4 h, and final crosslinking with tannic acid at room temperature for 2 h	0.1	- 86% optical transmittance at 600 nm - Low haze (around 17%) in the entire visible region - 100% UV-B radiation blocking	[80]
Treatment in alkaline NaClO_2_ solution at 80 °C for 18 h	Vacuum infiltration with an epoxy system at room temperature for 20 min, and final curing at 120 °C for 2 h	2	- 70% optical transmittance in the entire visible region	[81]
Treatment in an aqueous peroxyacetic acid solution at 80 °C for up to 4 h	Vacuum infiltration in a mixture of pre-polymerized methyl methacrylate and 4-vinylphenyl boronic acid for 30 min, and final UV-curing for 1 h	0.5 to 2	- 90% optical transmittance and 55% haze in the entire visible region	[82]

**Table 2:** Main optical properties of transparent pine woods.

Delignification Method	Infiltration Process and Polymerization/Curing	TW Thickness (mm)	Main Optical Outcomes	Ref.
None	In situ sulfonation in a Na_2_SO_3_ solution at different temperatures and times, compression at room temperature for up to 40 min, and final treatment at 150 °C for 30 min	0.095 to 0.17	- Thickness-dependent optical transmittance in the ~40–75% range at 800 nm	[83]
Treatment with acidic NaClO_2_ solution at 85 °C for up to 3 h	Vacuum infiltration with a low MW bisphenol A epoxy system at room temperature and subsequent lamination under pressure at room temperature	0.2	- Optical transmittance of 3-layer laminates between 45 and 65% in the entire visible range - Optical transmittance of 5-layer laminates between 35 and 55% in the entire visible range	[84]
Prior to delignification in a peracetic acid solution at 85 °C for 45 min, soaking of the native wood slices in an aqueous solution of 1,4-butanediol diglycidyl ether, degassing under vacuum for 20 min at room temperature, and crosslinking at 80 °C for 2 h	Hot compression at 105 °C under 15 MPa for 30 min	0.15	- 71% optical transmittance and 85% haze at 550 nm	[85]

**Table 3:** Main optical properties of transparent poplar woods.

Delignification Method	Infiltration Process and Polymerization/Curing	TW Thickness (mm)	Main Optical Outcomes	Ref.
Treatment with acidic NaClO_2_ solution at the boiling point for 3 h	Vacuum infiltration with poly(ethylene glycol) diacrylate and subsequent UV-curing for up to 40 s	0.15	- Over 90% optical transmittance and 70% haze in the entire visible region	[86]
Bleaching in a H_2_O_2_-based solution at 70 °C for 3 h	Vacuum infiltration with an epoxy system and curing at room temperature for 24 h	2	- 84% optical transmittance and 92% haze at 550 nm	[87]
Treatment with acidic NaClO_2_ solution at 80 °C for 4 h	Vacuum infiltration with pre-polymerized methyl methacrylate embedding a photochromic dye and curing at 75 °C for 5 h	0.65	- UV/visible switchable coloration - Over 80% optical transmittance in the entire visible region - UV-shielding	[88]
Treatment with acidic NaClO_2_ solution at 100 °C for 1.5 h	Infiltration with a flexible epoxy resin incorporating quantum dots, and curing at room temperature for 1 day	0.09	- Up to 90% optical transmittance in the entire visible range - Uniform luminescence under environmental exposure	[89]
Treatment with acidic NaClO_2_ solution at 80 °C for up to 6 h	Vacuum infiltration with pre-polymerized PMMA solution containing hydrochromic molecules	0.5	- Optical transmittance exceeding 80% in the entire visible range - Humidity-dependent color changes	[90]
Treatment with acidic NaClO_2_ solution at 85 °C for 6 h	Vacuum infiltration with a mixture of epoxy and urethane acrylates, UV-curing for 200 s	2	- Over 93% optical transmittance in the visible wavelength range	[91]

**Table 4:** Main optical properties of transparent basswoods.

Delignification Method	Infiltration Process and Polymerization/Curing	TW Thickness (mm)	Main Optical Outcomes	Ref.
Treatment with peracetic acid solution at 80 °C	Vacuum infiltration with a pre-polymerized methyl methacrylate solution for 3 h, and subsequent polymerization at 75 °C for 4 h	0.37	- About 85% optical transmittance and 72% haze at 550 nm	[92]
Treatment with acidic NaClO_2_ solution at 80 °C for 12 h	Compression at room temperature for 3 h to obtain densified films	0.094	- About 80% optical transmittance and 43% haze	[93]
Treatment with acidic NaClO_2_ solution at 85 °C for 2 h and subsequent dyeing in a red X-3B solution for 2 h	Vacuum infiltration with a UV-curable resin mixture for 24 h and subsequent UV-curing	0.5	- 81% optical transmittance in the visible range - Optical properties unaffected by the presence of the dye	[94]

**Table 5:** Main optical properties of transparent fir woods.

Delignification Method	Infiltration Process and Polymerization/Curing	TW Thickness (mm)	Main Optical Outcomes	Ref.
Treatment with acidic NaClO_2_ solution at 90 °C for 1.5 h	Impregnation with ethanolic solutions of silane coupling agent; vacuum infiltration with an epoxy resin system and subsequent curing at room temperature for up to 18 h	0.45	- Over 87% optical transmittance and less than 36% haze at 800 nm for the optimized TW	[95]
Treatment with acidic NaClO_2_ solution at 90 °C for 100 min	Vacuum infiltration with an epoxy resin system embedding chitosan and curing at room temperature for 24 h	1	- Around 74% optical transmittance and 87% haze at 550 nm for the TW containing 4 wt.% chitosan	[96]
Prior to treatment with acidic NaClO_2_ solution at 80 °C or with H_2_O_2_/acetic acid mixture at the same temperature, crosslinking with 1,4-butanediol diglycidyl ether	Vacuum infiltration with an epoxy resin system containing different amounts of dodecanol at 60 °C for 1 h, and curing at 75 °C for 5 h	1	- About 78% optical transmittance and 33% haze at 800 nm for the sample incorporating 70 wt.% dodecanol above the phase transition temperature - Around 48% optical transmittance and 86% haze at 800 nm for the sample incorporating 70 wt.% dodecanol below the phase transition temperature	[97]

**Table 6:** Main optical properties of transparent maple woods.

Delignification Method	Infiltration Process and Polymerization/Curing	TW Thickness (mm)	Main Optical Outcomes	Ref.
Treatment with acidic NaClO_2_ solution at 85 °C for 3 h	Vacuum infiltration with a UV-curable resin for 24 h and subsequent UV-curing for 5 min	0.4	- Around 89% optical transmittance and 52% haze at 800 nm	[98]
Treatment with acidic NaClO_2_ solution at 85 °C for 3 h	Vacuum infiltration with an epoxy resin for 3 h and subsequent curing	0.5	- Over 89% optical transmittance and about 57% haze	[99]
Treatment with acidic NaClO_2_ solution at 80 °C for 2 h	Infiltration of an ethanol dispersion of carbon quantum dots for 48 h, subsequent vacuum infiltration with an epoxy resin for 3 h, and final curing at room temperature for 24 h	0.5	- About 91% optical transmittance and over 70% haze in the entire visible range - Yellow fluorescence under 395 nm excitation	[100]

**Table 7:** Main optical properties of transparent bamboo materials.

Delignification Method	Infiltration Process and Polymerization/Curing	TW Thickness (mm)	Main Optical Outcomes	Ref.
Treatment in a H_2_O_2_-based alkaline solution at 70 °C	Vacuum infiltration with an epoxy resin and curing at room temperature	1.5	- 87% optical transmittance and 90% haze in the visible wavelength range	[101]
Treatment with acidic NaClO_2_ solution at 80–90 °C for up to 3 h	Vacuum infiltration with an epoxy resin, lamination in parallel or cross orientations, and final curing at 25 °C for 12 h	1.5	- About 82% optical transmittance and 72% haze within the visible wavelength range for 5 parallel laminated TW layers - Enhanced dimensional stability and UV resistance for cross-laminated structures	[102]
Pretreatment in NaOH solution at 80 °C, crosslinking with 1,2,3-propanetriol diglycidyl ether for 6 h, and final delignification with acidic NaClO_2_ solution at 80 °C	Infiltration with a flexible epoxy resin system and curing at 60 °C	1	- 80% optical transmittance and 72% haze	[103]
Crosslinking with 1,2,3-propanetriol diglycidyl ether in an alkaline ethanol solution, delignification in a 1 wt.% NaClO_2_ solution at 80 °C	Vacuum infiltration with an epoxy resin system incorporating photochromic microcapsules and curing at 60 °C for 24 h	0.9	- 78% optical transmittance and over 70% haze - Reversible and UV-responsive coloration	[104]
Treatment with acidic NaClO_2_ solution at 60 °C for 12 h	Infiltration with an epoxy resin system and subsequent parallel or cross lamination; final curing at room temperature for 12 h	0.6 to 1.5	- About 45% optical transmittance for low-density veneers - 10–12% optical transmittance for high-density veneers - Increased haze with increasing the fiber content	[105]

## Data Availability

No new data were created or analyzed in this study.

## Supplementary Materials

The following supporting information can be downloaded at: https://www.mdpi.com/article/10.3390/polym17243276/s1. References [110,111,112,113,114,115,116,117,118,119,120,121,122,123,124,125,126,127,128,129,130,131,132,133,134,135,136,137,138,139,140,141,142,143] are cited in the Supplementary Materials. Figure S1: Colorimetric parameters of Brazilian eucalyptus TW samples as a function of delignification time; Figure S2: Typical ASTM D1003 set-up for haze measurement of TW; Table S1: RI values at 589 nm of delignified balsa and birch wood samples; Table S2: Optical transmittance at 550 nm for different types of TW according to their composition and thickness; Table S3: Haze at 550 nm for different types of TW according to their composition and thickness.

## Abbreviations

The following abbreviations are used in this manuscript:

ASTM	American Society for Testing and Materials
CAN	Covalent adaptable network
CQD	Carbon quantum dot
CIE	Commission Internationale de l’Éclairage
CNT	Carbon nanotube
EDCP	Epoxy-based dynamic covalent polymer
EGDE	Ethylene glycol diglycidyl ether
EUA	Epoxy and urethane acrylates
FR	Flame retardant
Gel-TA	Gelatin-Tannic acid
HC	Hydrochromic molecule
HTW	Hydrochromic transparent wood
ILM	Immersion liquid method
ISO	International Standards Organization
MF	Melamine formaldehyde
MW	Molecular weight
NIR	Near-infrared
NP	Nanoparticle
NW	Nanowire
OB	Optical brightener
PAM	Polyacrylamide
PC	Photochromic pigment
PD	Photochromic dye
PEAG	Phosphate ester-polyethylene glycol
PEG	Polyethylene glycol
PEGDA	Poly(ethylene glycol) diacrylate
PLIMA	Poly(limonene acrylate)
PMMA	Poly(methyl methacrylate)
PTU	Polythiourethane
PVA	Polyvinyl alcohol
PVP	Polyvinyl pyrrolidone
PVPBA	Poly(4-vinylphenylboronic acid)
QD	Quantum dot
rGO	Reduced graphene oxide
RI	Refractive index
TA	Tannic acid
TEMPO	2,2,6,6-tetramethylpiperidine-1-oxyl radical
TMFP	Transport mean free path
TW	Transparent wood
UPR	Unsaturated polyester resin
UV	Ultraviolet
UVA	Ultraviolet absorber
WMO	Water-chromic molecule oxazolidines
WP	White phosphor
