# Supplementary material for: Toward the Optimization of the Optical Behavior of Transparent Wood: Current State of the Art and Perspectives
Source: Polymers (Basel). 2025 Dec 10;17(24):3276. doi: 10.3390/polym17243276 (PMC12737153; doi:10.3390/polym17243276)
Supplement: Supplementary file 1 [file polymers-17-03276-s001.zip › polymers-4019163-supplementary.pdf]

polymers

MDPI

Supplementary Materials

Toward the Optimization of the Optical Behavior of Transparent Wood: Current State of the art and Perspectives

Diego Pugliese [1] and Giulio Malucelli [2,*]

[1] Istituto Nazionale di Ricerca Metrologica (INRiM), Strada delle Cacce 91, 10135 Torino, Italy; d.pugliese@inrim.it
[2] Department of Applied Science and Technology, Politecnico di Torino, Viale Teresa Michel 5, 15121 Alessandria, Italy
* Correspondence: giulio.malucelli@polito.it; Tel.: +39-0131-229369

S1. Evaluating the Optical Behavior of Polymeric Systems

The main properties aimed at determining the optical behavior of TW, namely the refractive index (RI), the colorimetric response, the transmittance and the haze, will be detailed in the following Subsections.

S1.1. Refractive Index

Several methods are commonly adopted to measure the RI, including ellipsometry, interferometry, Abbe refractometry, total internal reflection, immersion liquid method (ILM), and prism coupling [110]. Each method offers distinct advantages in terms of accuracy, applicability, and compatibility with different material types, ranging from bulk solids and thin films to liquids.

Among the various methods available for determining the RI of solid materials, the ILM is particularly advantageous for TW due to its simplicity, non-destructive nature, and effectiveness in dealing with porous, heterogeneous structures. Unlike techniques such as ellipsometry or spectroscopic fitting, which may be limited by surface irregularities or require complex modeling, the ILM enables direct estimation of the effective RI by leveraging visual transparency [23]. The core principle involves immersing the material in a mixture of two liquids with known but different refractive indices. As the volume ratio of the two liquids is adjusted, a point is reached at which the RI of the liquid mixture matches that of the solid sample, rendering the composite visually transparent. The RI of the sample can then be calculated using Equation S1 [111]:

$$\mathrm{RI} = \frac{(V_a n_a + V_b n_b)}{(V_a + V_b)}, \quad \text{(S1)}$$

where V_a and V_b are the volumes, and n_a and n_b are the refractive indices of the two component liquids, respectively. This approach has been successfully applied to a wide range of materials, including minerals, wood powders, plastic fibers, and turbid media [23], and is especially well-suited for evaluating the optical matching between delignified wood templates and infiltrating polymers in TW fabrication.

In the development of TW, achieving superior optical transparency hinges on precisely matching the RI of the impregnating polymer with that of the wood's structural components—cellulose (RI = 1.525), hemicellulose (RI = 1.532), and lignin (RI = 1.610) [112]. Any mismatch in RI leads to increased light scattering, resulting in haze and reduced transparency. To mitigate this, a range of polymers has been employed, including poly(methyl methacrylate) (RI ~1.49), epoxy resin (RI ~1.50), polyvinylpyrrolidone (RI ~1.53), n-butyl methacrylate (RI ~1.50), polystyrene (RI ~1.59), dibutyl phthalate (RI ~1.52),

isobornyl methacrylate (RI ~1.48), diallyl phthalate (RI ~1.50), polyvinylcarbazole (RI ~1.68), and poly(acrylic acid) (RI ~1.45) [28]. Ensuring a close RI match between the polymer and the wood structure is essential for minimizing optical distortions and maximizing transparency, making RI optimization a key factor in the design of high-performance TW materials.

Chen et al. combined the ILM and a light transmission model based on Fresnel reflection/refraction theory to accurately determine the RI of delignified balsa and birch wood templates [111]. By immersing delignified samples in liquids of known RI and measuring light transmission, they were able to precisely estimate the RI of the delignified wood structure in both perpendicular and parallel fiber orientations at a wavelength of 589 nm (Table S1). Their findings reveal that the RI of delignified wood slightly varies based on fiber orientation, with perpendicular measurements yielding faintly higher values than parallel measurements. This anisotropy is likely due to the remaining cellulose microfibril alignment and structural changes occurring during the delignification process.

Table S1. RI values at 589 nm of delignified balsa and birch wood samples [111].

Wood type	Fiber direction	RI
Delignified balsa	Parallel	1.525 ± 0.008
Delignified balsa	Perpendicular	1.536 ± 0.006
Delignified birch	Parallel	1.529 ± 0.006
Delignified birch	Perpendicular	1.537 ± 0.005

S1.2. Colorimetric Response

The color measurements are commonly performed through precision colorimeters based on the Lab color space of the Commission Internationale de l'Éclairage (CIE). CIE-Lab defines L^* as lightness, which ranges from 100 (standard white) to 0 (black), and two chromatic parameters: a^* ($+a^*$ for redness, $-a^*$ for greenness) and b^* ($+b^*$ for yellowness, $-b^*$ for blueness) [108]. The total color change (ΔE^*) can be calculated using Equation S2:

$$\Delta E^* = \sqrt{[(\Delta L^*)^2 + (\Delta a^*)^2 + (\Delta b^*)^2]}, \tag{S2}$$

where ΔL^*, Δa^*, and Δb^* are the difference between initial and final values of L^*, a^*, and b^*, respectively.

Figure S1 shows the colorimetric values (L^*, a^*, b^*) of Brazilian eucalyptus TW samples at varying delignification times, i.e., 15, 30, 45, and 60 min. The figure clearly shows that as delignification time increases, the L^* value rises, indicating greater lightness, while a^* and b^* values drop, reflecting a reduction in redness and yellowness. This trend highlights the gradual whitening of wood due to lignin removal, confirming the visual impact of extended chemical treatment. However, a notable decrease in L^* is observed at the delignification time of 60 min, diverging from the expected upward trend. The underlying cause may stem from the complex interactions among wood's structural components: as delignification progresses, the progressive removal of lignin exposes cellulose and hemicellulose to potential chemical and physical alterations. This deviation from the anticipated brightening trend highlights the delicate balance required in wood modification processes and emphasizes the need for further investigation into the thresholds of effective delignification [39].

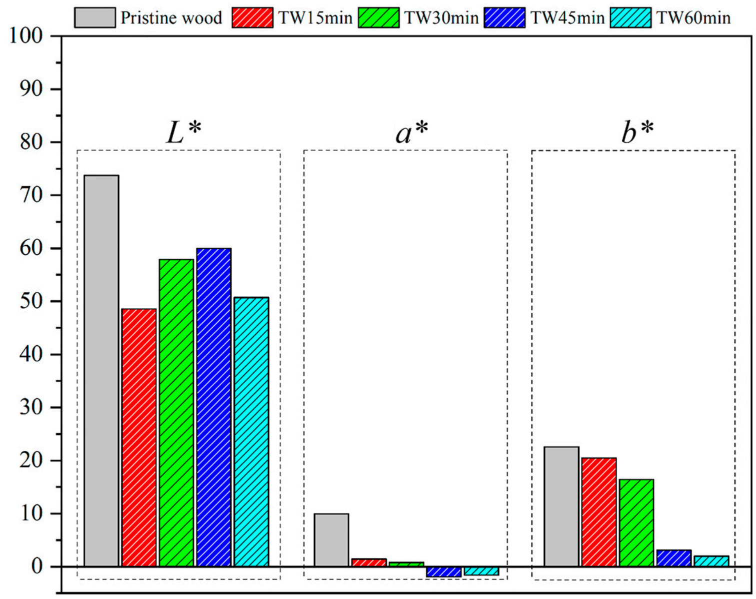

Figure S1. Colorimetric parameters of Brazilian eucalyptus TW samples as a function of delignification time. Reprinted from [39] under CC-BY-4.0 License.

S1.3. Optical Transmittance

Quantitative evaluation of transmittance, including both the specular and diffuse components, is typically conducted using an ultraviolet-visible-near-infrared (UV-Vis-NIR) spectrophotometer equipped with an integrating sphere. A 100% transmission reference spectrum (WHITE—W) is first obtained by directing the incoming beam into the integrating sphere via its entrance port, while ensuring that all secondary ports are sealed to suppress stray light interference. A 0% transmission baseline (DARK—D) is then recorded by deactivating the light source, thereby capturing the detector's background signal. The specimen is then positioned against the input port of the integrating sphere, ensuring complete coverage and perpendicular alignment with the incoming beam. Under identical measurement conditions, including parameters like spectral bandwidth, scanning speed, wavelength range, and beam alignment, the transmitted spectrum (SIGNAL—S), comprising both direct and scattered components, is acquired. Maintaining consistent settings across all measurements ensures reliability and eliminates potential biases arising from instrumental variability or optical misalignment. The overall transmittance is then calculated using Equation S3, providing a spectral profile of the sample's light transmission characteristics [113]:

$$T = \frac{S - D}{W - D} \quad \text{(S3)}$$

The transmittance of TW increases as lignin content is reduced. Additionally, it is influenced by several structural and processing factors, including type of polymer used, the botanical origin of the wood, cellulose volume fraction, fiber orientation, and sample thickness [24]. Table S2 collects some optical transmittance data at 550 nm for different types of TW in relation to their composition and thickness.

Table S2. Optical transmittance at 550 nm for different types of TW according to their composition and thickness.

Wood template	Infiltrating monomer/polymer	Thickness (mm)	Optical transmittance (%)	Ref.
Ailanthus	UPR	2	50	[114]
Aspen	Epoxy resin	2.5	~83	[115]
Balsa	Acrylic acid	0.55	85	[116]
Balsa	Acrylic resin	0.25	82.1	[117]
Balsa	Acrylic resin	0.49	82.5	[117]
Balsa	Acrylic resin	0.70	83.5	[117]
Balsa	Acrylic resin	1.10	81.1	[117]
Balsa	Epoxy resin	1	~90	[118]
Balsa	PAM	2	~72	[119]
Balsa	PAM + Ag NWs	2	~83	[119]
Balsa	PAM + CNTs	2	~84	[119]
Balsa	PAM + rGO	2	~85	[119]
Balsa	PLIMA	1.2	87	[106]
Balsa	PLIMA	2.0	80	[106]
Balsa	PLIMA	3.0	71	[106]
Balsa	PMMA	0.7	90	[113]
Balsa	PMMA	1.2	85	[113]
Balsa	PMMA	3.7	40	[113]
Balsa	PVA	0.8	91	[120]
Balsa	Thiol-ene	1.1	87	[121]
Bamboo	Epoxy resin	0.6	82.4	[103]
Bamboo	Epoxy resin	1.0	79.1	[103]
Bamboo	Epoxy resin	1.5	76.9	[103]
Basswood	Epoxy resin	5	87	[122]
Basswood	PMMA	1	86	[123]
Basswood	PVP	1	90	[124]
Beech	PMMA	0.1	70	[125]
Beech	PMMA	0.3	30	[125]
Beech	PMMA	0.6	18	[125]
Beech	PMMA	0.7	15	[125]
Birch	MF	1.2	74	[63]
Birch	PEG/PMMA	0.5	84	[126]
Birch	PEG/PMMA	1.5	68	[126]
Birch	PLIMA	0.7	88	[106]
Birch	PMMA	0.7	94	[127]
Birch	PMMA	1.1	74	[128]
Birch	PMMA	1.3	70	[129]
Birch	PMMA	1.5	64	[40]
Birch	Thiol-ene	1.2	89	[130]
Brazilian eucalyptus	PMMA	0.6	71.68	[39]
Cathay poplar	PMMA	0.5	90.4	[131]
Douglas fir	Epoxy resin	2	~80	[35]
Japanese cypress	UV-curable resin	0.46	62	[132]
Larch	Epoxy resin	2.5	~54	[115]
Mahogany	Epoxy resin	0.5	80	[133]
Melia	Epoxy resin	2	~76.7	[87]
Melia	Epoxy resin	2	77.75	[134]
Melia	Epoxy resin	4	55.94	[134]
Melia	Epoxy resin	6	36.11	[134]

Melia	Epoxy resin	8	31.22	[134]
Melia	Epoxy resin + UVA	2	83.4	[108]
New Zealand pine	PMMA	0.5	~9	[135]
Paulownia	Epoxy resin/EGDE	20	89	[136]
Poplar	Epoxy resin	2	83.53	[137]
Poplar	Epoxy resin	2	83.53	[134]
Poplar	Epoxy resin	4	58.15	[134]
Poplar	Epoxy resin	6	46.40	[134]
Poplar	Epoxy resin	8	37.09	[134]
Poplar	Epoxy resin + UVA	2	75.5	[108]
Poplar	PMMA	0.5	86.1	[138]
Poplar	PMMA	1	92	[139]
Poplar	PVA	1	80	[140]
Rubberwood	Epoxy resin	1.5	64	[141]
Rubberwood	UPR	1.5	64	[141]
Rubberwood	UPR	2	54	[114]
Silver birch	1-dodecanol/PLIMA	0.5	86	[142]
Silver oak	Epoxy resin	1	73	[107]
Silver oak	Epoxy resin	2	~72.8	[87]
Silver oak	Epoxy resin	2	72.84	[134]
Silver oak	Epoxy resin	4	67.44	[134]
Silver oak	Epoxy resin	6	48.83	[134]
Silver oak	Epoxy resin	8	31.71	[134]
Silver oak	Epoxy resin + OB	1	82	[107]
White spruce	Epoxy resin	2.5	~37	[115]

S1.4. Haze

Haze quantifies transparency in imaging contexts, such as how clearly an ambient scene can be observed through TW. According to the American Society for Testing and Materials (ASTM) D1003 [143], haze is defined as the percentage of transmitted light that is scattered at angles greater than 2.5° from the incident beam direction. Measurements are typically performed using an integrating sphere set-up, involving four key steps (Figure S2) [45]: (1) T_1—reference transmittance with no sample and a white standard; (2) T_2—light transmitted through the sample with a white standard; (3) T_3—instrumental scattering with no sample and a light trap; and (4) T_4—total scattered light with both sample and light trap. The haze value is then calculated using Equation S4:

$$\text{Haze} = \frac{T_d}{T_t} = \left(\frac{T_4}{T_2} - \frac{T_3}{T_1}\right) \times 100\% \quad \text{(S4)}$$

where T_d is the diffused transmittance and T_t is the total transmittance. Here, T_3/T_1 accounts for the instrumental scattering error.

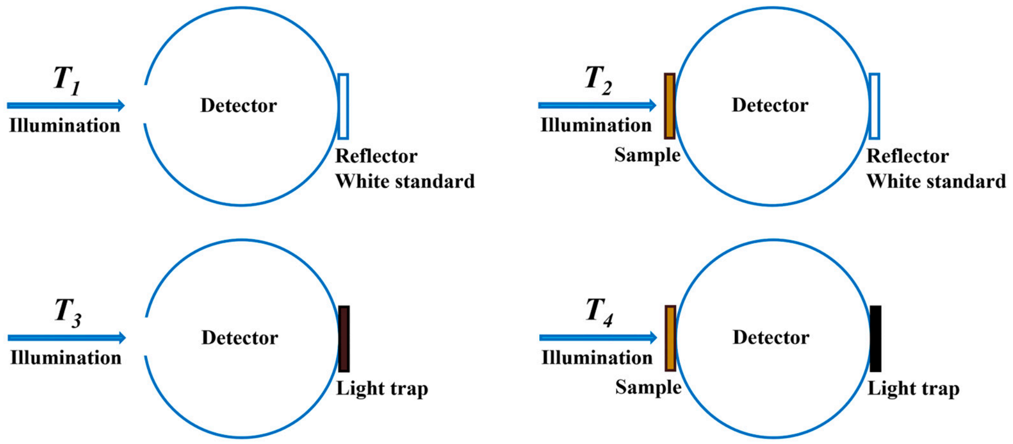

Figure S2. Typical ASTM D1003 set-up for haze measurement of TW.

Haze in TW is influenced by several factors including anisotropy, thickness, and transport mean free path (TMFP), as well as the intrinsic microstructure of the wood (rich of micro-curvatures, bumps, and cavities, among others), which increase RI variations and light scattering [40]. Additionally, the RI mismatch between the delignified wood template and infiltrating polymer plays a significant role [24]. High haze values make TW especially suitable for applications in privacy windows, smart building facades, and light diffusers in solar energy systems, where controlled light scattering is advantageous [21]. The haze values of different TW samples, varying in wood species, types of infiltrated monomers/polymers, and sample thickness, are summarized in Table S3.

Table S3. Haze at 550 nm for different types of TW according to their composition and thickness.

Wood template	Infiltrating monomer/polymer	Thickness (mm)	Haze (%)	Ref.
Ailanthus	UPR	2	94	[114]
Balsa	Acrylic acid	0.55	85	[116]
Balsa	Acrylic resin	0.25	40.0	[117]
Balsa	Acrylic resin	0.49	46.3	[117]
Balsa	Acrylic resin	0.70	52.7	[117]
Balsa	Acrylic resin	1.10	58.5	[117]
Balsa	Epoxy resin	1	~60	[118]
Balsa	PLIMA	1.2	46	[106]
Balsa	PLIMA	2.0	62	[106]
Balsa	PLIMA	3.0	65	[106]
Balsa	PMMA	0.7	50	[113]
Balsa	PMMA	1.2	71	[113]
Balsa	PMMA	3.7	80	[113]
Balsa	PVA	0.8	15	[120]
Balsa	Thiol-ene	1.1	47	[121]
Basswood	Epoxy resin	5	90	[122]
Basswood	PMMA	1	70	[123]
Basswood	PVP	1	80	[124]
Beech	PMMA	0.3	18	[125]
Beech	PMMA	0.6	40	[125]
Beech	PMMA	0.7	49	[125]
Birch	MF	1.2	66	[63]
Birch	PEG/PMMA	0.5	74	[126]
Birch	PEG/PMMA	1.5	77	[126]
Birch	PLIMA	0.7	49	[106]

Birch	PMMA	0.7	31	[127]
Birch	PMMA	1.1	92	[128]
Birch	PMMA	1.3	70	[129]
Birch	PMMA	1.5	80	[40]
Birch	Thiol-ene	1.2	61	[130]
Douglas fir	Epoxy resin	2	~93	[35]
Japanese cypress	UV-curable resin	0.46	72.3	[132]
Mahogany	Epoxy resin	0.5	37	[133]
Melia	Epoxy resin	2	84.9	[87]
Paulownia	Epoxy resin/EGDE	20	97	[136]
Poplar	Epoxy resin	2	94.70	[137]
Poplar	PMMA	1	56	[139]
Poplar	PVA	1	90	[140]
Rubberwood	Epoxy resin	1.5	88	[141]
Rubberwood	UPR	1.5	90	[141]
Rubberwood	UPR	2	94	[114]
Silver birch	1-dodecanol/PLIMA	0.5	73	[142]
Silver oak	Epoxy resin	1	84	[107]
Silver oak	Epoxy resin	2	90.6	[87]
Silver oak	Epoxy resin + OB	1	90	[107]

S2. Cross-Analysis of the Optical Data Collected in Tables S1 and S2

S2.1. Thickness Dependence

As shown in Tables S1 and S2, there is a clear inverse correlation between sample thickness and optical transmittance, which is characteristic of multiple-scattering media. For example, in balsa-PMMA TWs, transmittance decreases sharply from 90% at 0.7 mm to 40% at 3.7 mm, while haze rises correspondingly from 50 to 80%. A similar trend is observed in melia-epoxy TWs, where transmittance drops from approximately 78% at 2 mm to around 31% at 8 mm. This attenuation is due to cumulative RI mismatches and increased internal interfaces along the light path, which cause enhanced forward scattering. Thus, thinner veneers (≤1 mm) consistently deliver the highest transmittance and lowest haze, approaching the theoretical optical limits set by RI matching between cellulose and infiltrated polymer matrices.

S2.2. Influence of Wood Species

Significant species-dependent variation exists due to differences in cell wall porosity, fiber orientation, and intrinsic RI anisotropy. Balsa, for example, demonstrates the highest optical performance among low-density woods. Transmittance often exceeds 85-90% for 0.5-1 mm samples, and haze remains moderate (approximately from 50 to 70%), depending on the infiltrated monomer(s). Its open cellular architecture favors efficient monomer infiltration and minimal residual voids. Conversely, birch and basswood are denser with thicker cell walls and still achieve high transmittance (85-94%), though usually at smaller thicknesses (≤1 mm). Poplar and pine display good optical uniformity, achieving >80% transmittance and haze values near 90%, though optical anisotropy increases with radial versus tangential orientation. Bamboo and melia are characterized by higher vascular density and silica or resin inclusions. They exhibit relatively lower optical transmittance (between 75 and 80%) and higher haze at similar thicknesses due to light scattering from heterogeneous fiber bundles. Overall, species with low density, uniform lumen distribution, and isotropic grain orientation (e.g., balsa and birch) provide the best optical balance between clarity and scattering.

S2.3. Correlations with Monomer Type

The infiltrating polymer strongly influences transmittance and haze through its RI, cross-linking density, and ability to wet the delignified cell walls. PMMA (RI ~ 1.49) and epoxy resins (RI ~ 1.50) have the most favorable RI matching with cellulose (RI ~ 1.53). This results in high transmittance (up to 94%) and moderate to high haze (between 50 and 90%). PVA leads to exceptionally high transmittance (91%) but low haze (15%), due to its hydrogen-bonding compatibility and smooth interfaces. UPR and MF resins typically show high haze (>90%) and low transmittance (50-70%), indicating incomplete index matching and higher scattering. Conversely, hybrid or functionalized systems, such as PLIMA and PEG/PMMA blends, demonstrate balanced optical behavior (transmittance: 80-88%; haze: 45-65%), suggesting that these bio-based monomers can rival petrochemical matrices when RI and infiltration kinetics are optimized. Lastly, the incorporation of fillers (e.g., Ag nanowires, CNTs, TiO_2, rGO) into the infiltrated monomer(s) slightly increases haze but can improve transmittance if the filler raises the matrix RI toward that of delignified/bleached wood template.

S2.4. Combined Trends

By comparing the data in Tables S1 and S2, the following outcomes stand out:

- high optical transmittance (>85%) is consistently achieved with a thickness below 1 mm, low-density species (balsa, birch, and poplar), and PMMA/epoxy/PVA infiltration.

- high haze (>80%), which is suitable for diffusive glazing, correlates with thicker samples (>2 mm), denser species (basswood, pine, and fir), and resins with poor RI matching (UPR and MF).

- in most systems, transmittance and haze are positively correlated up to a threshold of about 80% transmittance and 70-90% haze. Beyond this threshold, increasing density or thickness causes simultaneous losses in both metrics due to excessive scattering phenomena.

In conclusion, from an optical design standpoint, maximizing transparency of TWs requires minimizing scattering through the following strategies: (i) finding a precise RI matching between the resin and cellulose; (ii) achieving microstructural uniformity via thorough infiltration and densification; and (iii) controlling thickness to limit the optical path length.

Abbreviations

The following abbreviations are used in this manuscript:

ASTM	American Society for Testing and Materials
CIE	Commission Internationale de l'Éclairage
CNT	Carbon nanotube
EGDE	Ethylene glycol diglycidyl ether
ILM	Immersion liquid method
MF	Melamine formaldehyde
NW	Nanowire
OB	Optical brightener
PAM	Polyacrylamide
PEG	Polyethylene glycol
PLIMA	Poly(limonene acrylate)
PMMA	Poly(methyl methacrylate)
PVA	Polyvinyl alcohol
PVP	Polyvinyl pyrrolidone
rGO	Reduced graphene oxide
RI	Refractive index
TMFP	Transport mean free path

TW	Transparent wood
UPR	Unsaturated polyester resin
UV-Vis-NIR	Ultraviolet-Visible-Near-infrared
UVA	Ultraviolet absorber

References

21. Li, Y.; Fu, Q.; Yang, X.; Berglund, L. Transparent wood for functional and structural applications. *Phil. Trans. R. Soc. A* **2018**, *376*, 20170182.

23. Chutturi, M.; Gillela, S.; Yadav, S.M.; Wibowo, E.S.; Sihag, K.; Rangppa, S.M.; Bhuyar, P.; Siengchin, S.; Antov, P.; Kristak, L.; Sinha, A. A comprehensive review of the synthesis strategies, properties, and applications of transparent wood as a renewable and sustainable resource. *Sci. Total Environ.* **2023**, *864*, 161067.

24. Zhang, J.; Koubaa, A.; Tao, Y.; Li, P.; Xing, D. The emerging development of transparent wood: materials, characteristics, and applications. *Curr. For. Rep.* **2022**, *8*, 333–345.

28. Jele, T.B.; Andrew, J.; John, M.; Sithole, B. Engineered transparent wood composites: a review. *Cellulose* **2023**, *30*, 5447–5471.

35. Mi, R.; Chen, C.; Keplinger, T.; Pei, Y.; He, S.; Liu, D.; Li, J.; Dai, J.; Hitz, E.; Yang, B.; Burgert, I.; Hu, L. Scalable aesthetic transparent wood for energy efficient buildings. *Nat. Commun.* **2020**, *11*, 3836.

39. Barbosa, K.T.; Cardoso, G.V.; Acosta, A.P.; Aramburu, A.B.; De Avila Delucis, R.; Gatto, D.A.; Labidi, J.; Beltrame, R. Unveiling the potential of Brazilian eucalyptus for transparent wood manufacturing via the kraft pulping process as a future building material. *Forests* **2024**, *15*, 1544.

40. Li, Y.; Fu, Q.; Rojas, R.; Yan, M.; Lawoko, M.; Berglund, L. Lignin-retaining transparent wood. *ChemSusChem* **2017**, *10*, 3445–3451.

45. Li, Y.; Vasileva, E.; Sychugov, I.; Popov, S.; Berglund, L. Optically transparent wood: Recent progress, opportunities, and challenges. *Adv. Opt. Mater.* **2018**, *6*, 1800059.

63. Samanta, P.; Samanta, A.; Montanari, C.; Li, Y.; Maddalena, L.; Carosio, F.; Berglund, L.A. Fire-retardant and transparent wood biocomposite based on commercial thermoset. *Composites, Part A* **2022**, *156*, 106863.

87. Bisht, P.; Pandey, K.K.; Srinivas, G. Physicochemical characterization and thermal behaviour of transparent wood composite. *Mater. Today Commun.* **2022**, *31*, 103767.

103. Wang, K.; Peng, H.; Gu, Q.; Zhang, X.; Liu, X.; Dong, Y.; Cai, Y.; Li, Y.; Li, J. Scalable, large-size, and flexible transparent bamboo. *Chem. Eng. J.* **2023**, *451*, 138349.

106. Montanari, C.; Ogawa, Y.; Olsén, P.; Berglund, L.A. High performance, fully bio-based, and optically transparent wood biocomposites. *Adv. Sci.* **2021**, *8*, 2100559.

107. Anish, M.C.; Subba Rao, A.N.; Nair, S.; Nagarajappa, G.B.; Pandey, K.K. Luminescent transparent wood from a woody cellulosic template treated with an optical brightener. *J. Appl. Polym. Sci.* **2023**, *140*, e54028.

108. Bisht, P.; Barshilia, H.C.; Pandey, K.K. Effect of natural weathering on optical properties of transparent wood composite. *Polym. Degrad. Stab.* **2024**, *221*, 110674.

110. Chiu, M.-H.; Lai, C.-W.; Wang, S.-F.; Su, D.-C.; Chang, S. Phase geographical map for determining the material type of a right-angle prism. *Appl. Opt.* **2006**, *45*, 6781–6784.

111. Chen, H.; Montanari, C.; Yan, M.; Popov, S.; Li, Y.; Sychugov, I.; Berglund, L.A. Refractive index of delignified wood for transparent biocomposites. *RSC Adv.* **2020**, *10*, 40719–40724.

112. Vasileva, E.; Chen, H.; Li, Y.; Sychugov, I.; Yan, M.; Berglund, L.; Popov, S. Light scattering by structurally anisotropic media: A benchmark with transparent wood. *Adv. Opt. Mater.* **2018**, *6*, 1800999.

113. Li, Y.; Fu, Q.; Yu, S.; Yan, M.; Berglund, L. Optically transparent wood from a nanoporous cellulosic template: Combining functional and structural performance. *Biomacromolecules* **2016**, *17*, 1358–1364.

114. Anish, M.C.; Pandey, K.K.; Kumar, R. Transparent wood composite prepared from two commercially important tropical timber species. *Sci. Rep.* **2023**, *13*, 14915.

115. Bradai, H.; Koubaa, A.; Zhang, J.; Demarquette, N.R. Effect of wood species on lignin-retaining high-transmittance transparent wood biocomposites. *Polymers* **2024**, *16*, 2493.

116. Bi, Z.; Li, T.; Su, H.; Ni, Y.; Yan, L. Transparent wood film incorporating carbon dots as encapsulating material for white light-emitting diodes. *ACS Sustainable Chem. Eng.* **2018**, *6*, 9314–9323.

117. Wang, S.; Li, L.; Zha, L.; Koskela, S.; Berglund, L.A.; Zhou, Q. Wood xerogel for fabrication of high-performance transparent wood. *Nat. Commun.* **2023**, *14*, 2827.

118. Xia, Q.; Chen, C.; Li, T.; He, S.; Gao, J.; Wang, X.; Hu, L. Solar-assisted fabrication of large-scale, patternable transparent wood. *Sci. Adv.* **2021**, *7*, eabd7342.
119. Zhang, B.; Cui, J.; He, D.; Zhang, J.; Yang, L.; Zhu, W.; Lv, H. Transparent electromagnetic absorption film derived from the biomass derivate. *J. Mater. Sci. Technol.* **2024**, *185*, 98–106.
120. Mi, R.; Li, T.; Dalgo, D.; Chen, C.; Kuang, Y.; He, S.; Zhao, X.; Xie, W.; Gan, W.; Zhu, J.; Srebric, J.; Yang, R.; Hu, L. A clear, strong, and thermally insulated transparent wood for energy efficient windows. *Adv. Funct. Mater.* **2020**, *30*, 1907511.
121. Samanta, A.; Chen, H.; Samanta, P.; Popov, S.; Sychugov, I.; Berglund, L.A. Reversible dual-stimuli-responsive chromic transparent wood biocomposites for smart window applications. *ACS Appl. Mater. Interfaces* **2021**, *13*, 3270–3277.
122. Li, H.; Guo, X.; He, Y.; Zheng, R. A green steam-modified delignification method to prepare low-lignin delignified wood for thick, large highly transparent wood composites. *J. Mater. Res.* **2019**, *34*, 932–940.
123. Li, Y.; Cheng, M.; Jungstedt, E.; Xu, B.; Sun, L.; Berglund, L. Optically transparent wood substrate for perovskite solar cells. *ACS Sustainable Chem. Eng.* **2019**, *7*, 6061–6067.
124. Zhu, M.; Li, T.; Davis, C.S.; Yao, Y.; Dai, J.; Wang, Y.; AlQatari, F.; Gilman, J.W.; Hu, L. Transparent and haze wood composites for highly efficient broadband light management in solar cells. *Nano Energy* **2016**, *26*, 332–339.
125. Yaddanapudi, H.S.; Hickerson, N.; Saini, S.; Tiwari, A. Fabrication and characterization of transparent wood for next generation smart building applications. *Vacuum* **2017**, *146*, 649–654.
126. Montanari, C.; Li, Y.; Chen, H.; Yan, M.; Berglund, L.A. Transparent wood for thermal energy storage and reversible optical transmittance. *ACS Appl. Mater. Interfaces* **2019**, *11*, 20465–20472.
127. Li, Y.; Yang, X.; Fu, Q.; Rojas, R.; Yan, M.; Berglund, L. Towards centimeter thick transparent wood through interface manipulation. *J. Mater. Chem. A* **2018**, *6*, 1094–1101.
128. Samanta, P.; Samanta, A.; Maddalena, L.; Carosio, F.; Gao, Y.; Montanari, C.; Nero, M.; Willhammar, T.; Berglund, L.A.; Li, Y. Coloration and fire retardancy of transparent wood composites by metal ions. *ACS Appl. Mater. Interfaces* **2023**, *15*, 58850–58860.
129. Jungstedt, E.; Montanari, C.; Östlund, S.; Berglund, L. Mechanical properties of transparent high strength biocomposites from delignified wood veneer. *Composites, Part A* **2020**, *133*, 105853.
130. Höglund, M.; Johansson, M.; Sychugov, I.; Berglund, L.A. Transparent wood biocomposites by fast UV-curing for reduced light-scattering through wood/thiol–ene interface design. *ACS Appl. Mater. Interfaces* **2020**, *12*, 46914–46922.
131. Gan, W.; Gao, L.; Xiao, S.; Zhang, W.; Zhan, X.; Li, J. Transparent magnetic wood composites based on immobilizing Fe_3O_4 nanoparticles into a delignified wood template. *J. Mater. Sci.* **2017**, *52*, 3321–3329.
132. Tong, K.; Zhang, H.; Zhang, Y.; Liu, J.; Wu, Y. An ultraviolet radiation protective transparent wood film retained natural wood texture and tactile properties. *Polymer* **2024**, *311*, 127560.
133. Popović, J.; Svrzić, S.; Gajić, M.; Maletić, S.; Dodevski, V.; Djiporović-Momčilović, M.; Krstić, S.; Popović, M. Light transmittance of mahogany wood treated with 20% hydrogen peroxide solution. *BioResources* **2022**, *17*, 5919–5935.
134. Bisht, P.; Pandey, K.K. Optical and mechanical properties of multilayered transparent wood. *Mater. Today Commun.* **2024**, *38*, 107871.
135. Wu, Y.; Zhou, J.; Yang, F.; Wang, Y.; Wang, J.; Zhang, J. A strong multilayered transparent wood with natural wood color and texture. *J. Mater. Sci.* **2021**, *56*, 8000–8013.
136. Cai, H.; Wang, Z.; Xie, D.; Zhao, P.; Sun, J.; Qin, D.; Cheng, F. Flexible transparent wood enabled by epoxy resin and ethylene glycol diglycidyl ether. *J. For. Res.* **2021**, *32*, 1779–1787.
137. Bisht, P.; Pandey, K.K.; Barshilia, H.C. Photostable transparent wood composite functionalized with an UV-absorber. *Polym. Degrad. Stab.* **2021**, *189*, 109600.
138. Gan, W.; Xiao, S.; Gao, L.; Gao, R.; Li, J.; Zhan, X. Luminescent and transparent wood composites fabricated by poly(methyl methacrylate) and γ-Fe_2O_3@YVO_4:Eu^{3+} nanoparticle impregnation. *ACS Sustainable Chem. Eng.* **2017**, *5*, 3855–3862.
139. Wang, X.; Zhan, T.; Liu, Y.; Shi, J.; Pan, B.; Zhang, Y.; Cai, L.; Shi, S.Q. Large-size transparent wood for energy-saving building applications. *ChemSusChem* **2018**, *11*, 4086–4093.
140. Subba Rao, A.N.; Nagarajappa, G.B.; Nair, S.; Chathoth, A.M.; Pandey, K.K. Flexible transparent wood prepared from poplar veneer and polyvinyl alcohol. *Compos. Sci. Technol.* **2019**, *182*, 107719.
141. Anish, M.C.; Pandey, K.K.; Kumar, R. Preparation and characterization of unsaturated polyester infused transparent wood composites. *Eur. J. Wood Wood Prod.* **2024**, *82*, 503–513.
142. Montanari, C.; Chen, H.; Lidfeldt, M.; Gunnarsson, J.; Olsén, P.; Berglund, L.A. Sustainable thermal energy batteries from fully bio-based transparent wood. *Small* **2023**, *19*, 2301262.

143. ISO 14782:2021(en) Plastics — Determination of Haze for Transparent Materials. Available online: https://www.iso.org/obp/ui/en/#iso:std:iso:14782:ed-2:v1:en:en (accessed on 16 April 2025).

Disclaimer/Publisher's Note: The statements, opinions and data contained in all publications are solely those of the individual author(s) and contributor(s) and not of MDPI and/or the editor(s). MDPI and/or the editor(s) disclaim responsibility for any injury to people or property resulting from any ideas, methods, instructions or products referred to in the content.
